# Association of urinary post-translationally modified fetuin-A fragments with diabetic kidney disease risk stratification in Japanese patients with type 2 diabetes

**DOI:** 10.1371/journal.pone.0353032

**Published:** 2026-07-02

**Authors:** Toshiko Mori, Hiroyuki Ito, Taiki Kozasa, Chizuko Yukawa, Suzuko Matsumoto, Hideyuki Inoue, Shinichi Antoku

**Affiliations:** 1 Department of Diabetes, Metabolism and Kidney Disease, Edogawa Hospital, Tokyo, Japan; 2 Clinical Test Management Division, PreMedica Inc., Tokyo, Japan; 3 Laboratory Department, Edogawa Hospital, Tokyo, Japan; International University of Health and Welfare, School of Medicine, JAPAN

## Abstract

**Aims:**

Conventional biomarkers such as estimated glomerular filtration rate (eGFR) and urinary albumin-to-creatinine ratio (uACR) primarily reflect glomerular damage and often fail to detect early tubular injury. Consequently, patients with “non-albuminuric diabetic kidney disease (DKD)” may be overlooked. This study evaluated the independent association between urinary post-translationally modified fetuin-A fragments (uPTM-FetA) and DKD risk stratification in Japanese patients with type 2 diabetes.

**Methods:**

We conducted a cross-sectional study of 219 outpatients with type 2 diabetes between November 2023 and February 2024 at Edogawa Hospital. First-morning urine samples were analyzed for uPTM-FetA and urinary liver-type fatty acid-binding protein (uL-FABP) using enzyme-linked immunosorbent assays. DKD risk was classified into four categories based on the KDIGO guidelines. The association between uPTM-FetA and higher DKD-risk (categories 2 + 3 + 4) was assessed using multiple logistic regression and restricted cubic spline (RCS) analyses, validated by bootstrapping.

**Results:**

The optimal cutoff value for uPTM-FetA was determined to be 11.76 ng/mgCr. Multivariable analysis adjusted for potential confounders revealed that high uPTM-FetA levels were significantly and independently associated with DKD-risk categories 2 + 3 + 4 (adjusted odds ratio: 3.88; 95% CI: 2.02–7.45; P < 0.01). RCS analysis indicated a significant non-linear association (P = 0.04). Notably, high uPTM-FetA was detected in 38.8% of patients with normoalbuminuria and 42.0% of those with preserved eGFR. A striking discrepancy was observed compared to uL-FABP: while high uL-FABP was completely absent (0.0%) in patients within the low-to-moderate risk categories (categories 1 and 2), high uPTM-FetA was observed in 34.0% and 60.8% of these patients, respectively.

**Conclusions:**

uPTM-FetA is independently associated with DKD severity and is elevated in a substantial proportion of patients with early-stage disease where conventional markers remain normal. Unlike uL-FABP, which increases predominantly in advanced stages, uPTM-FetA appears to identify tubular stress earlier. Thus, uPTM-FetA serves as a valuable complementary biomarker to uACR for refining DKD risk stratification.

## Introduction

As the number of patients with type 2 diabetes continues to rise globally, diabetic kidney disease (DKD) has become the leading cause of chronic kidney disease (CKD) and end-stage renal disease (ESRD) [1,2]. Because the progression of DKD can be slowed with timely intervention, the accurate identification of patients at high risk for declining renal function is crucial. This approach may help to reduce the number of patients who require renal replacement therapy.

The estimated glomerular filtration rate (eGFR) and urinary albumin-to-creatinine ratio (uACR) were the primary biomarkers used to assess CKD severity. However, the eGFR can exhibit substantial intra-individual variability and is influenced by muscle mass, making it a suboptimal indicator in certain clinical contexts, particularly in the early stages of DKD [3,4].

Furthermore, clinical heterogeneity in DKD progression has become increasingly apparent. Approximately 20% of patients with type 2 diabetes and normoalbuminuric DKD progress to advanced CKD or ESRD within 10 years [5]. Similarly, a study conducted at the Steno Diabetes Center found that approximately 20% of diabetic patients without albuminuria never develop proteinuria before reaching ESRD [6]. These findings suggest that albuminuria alone may not fully capture the diverse pathophysiological mechanisms of DKD, specifically those unrelated to glomerular barrier dysfunction.

One possible reason for the limitations of eGFR and uACR is that they may not fully capture the early pathophysiological changes occurring before overt functional decline. There is a critical need for biomarkers that can identify renal stress at an earlier stage [7]. Ideally, such biomarkers would reflect specific molecular mechanisms driving disease progression. Although uACR serves as a key marker for diagnosing, monitoring, and managing DKD, its sensitivity, and specificity have limitations, as its levels can increase in other glomerular diseases. Additionally, up to 40% of patients with type 2 diabetes may present with normal uACR despite having biopsy-confirmed renal lesions [8].

Renal biopsy remains the gold standard for diagnosing the specific pathology of DKD; however it is an invasive procedure associated with potential complications and is not feasible for routine screening. Therefore, there is a critical need for non-invasive biomarkers that can complement uACR and eGFR to refine risk stratification and reflect distinct pathological pathways.

Traditional diagnostic frameworks for DKD have focused primarily on glomerular injury, with uACR serving as the cardinal marker [9]. However, it is increasingly recognized that tubular injury, characterized by proximal tubular cell stress, hypoxia, and interstitial fibrosis, that plays a critical and often independent role in the pathogenesis and progression of DKD [10,11]. While albuminuria reflects glomerular damage, it may not sufficiently capture tubular pathology, leading to the clinical challenge of identifying patients with “non-albuminuric DKD” which remain high risk [8,12,13]. The Kidney Disease: Improving Global Outcomes (KDIGO) risk grid, integrating eGFR and uACR, provides a composite clinical framework that reflects overall prognosis more accurately than either marker alone [14]. Nevertheless, incorporating biomarkers that specifically reflect tubular health could further enhance this risk assessment.

Fetuin-A, a 64-kDa glycoprotein encoded by the AHSG gene, is a strong candidate in this context. While primarily secreted by the liver as a mediator of insulin resistance and inflammation [15–17], its role in renal dysfunction is increasingly recognized [18,19]. Crucially, urinary post-translationally modified fetuin-A (uPTM-FetA) has emerged as a distinct biomarker from its circulating form. Unlike circulating fetuin-A, uPTM-FetA is specifically produced by injured proximal tubule epithelial cells (PTECs) in response to stressors such as hypoxia [20,21]. This localized production suggests a mechanistic pathway distinct from both albuminuria and other tubular markers, such as urinary liver-type fatty acid-binding protein (uL-FABP), which primarily reflects fatty acid metabolism stress [22,23].

While previous studies have identified uPTM-FetA as a potential biomarker for DKD in specific populations [24,25], its relationship with the established clinical risk categories remains to be fully elucidated. Therefore, the aim of this cross-sectional study was to evaluate the association between uPTM-FetA levels and KDIGO-defined DKD-risk categories in Japanese patients with type 2 diabetes. Specifically, we investigated whether uPTM-FetA provides complementary information to established markers such as uACR, thereby enhancing the stratification of DKD risk.

## Materials and methods

### Study design and patients

This cross-sectional study was conducted between November 16, 2023, and February 26, 2024, at the Department of Diabetes, Metabolism, and Kidney Disease, Edogawa Hospital, Japan. Inclusion criteria were adult Japanese outpatients with type 2 diabetes. Exclusion criteria were as follows: pregnancy; a history of dementia, alcoholism, or drug addiction; presence of malignant tumors; acute or chronic infections; and CKD diagnosed prior to the onset of diabetes.

The attending physician provided verbal explanations of the study purpose, procedures, and risks. Subsequently, both verbal and written informed consent were obtained. Written consent forms were signed by the participants and collected by the investigators. Verbal consent was documented in the presence of the attending physician, who served as a witness.

### CKD classification and risk assessment

The eGFR was calculated using the Japanese equation as follows [26]:


$$\begin{array}{c} \text{eGFR }(\text{mL}/\text{min}/\text{1}.\text{73 }{\text{m}}^{\text{2}})=\text{ 194 }\times \text{serum }{\text{Cr}}^{-\text{1}.\text{094}}\times {\text{age}}^{-\text{0}.\text{287}}\times \text{0}.\text{739 }\left(\text{if female}\right) \end{array}$$

Albuminuria and GFR categories were classified based on the KDIGO guidelines [14]. Albuminuria was categorized as A1 (uACR < 30 mg/gCr), A2 (30–299 mg/gCr), or A3 (≥300 mg/gCr). GFR was categorized as G1 (≥90 mL/min/1.73 m²), G2 (60–89), G3a (45–59), G3b (30–44), G4 (15–29), and G5 (<15).

DKD risk was stratified into four categories based on the KDIGO prognosis risk heat map [14]:

(1) Category 1 (low risk, green): GFR ≥ 60 mL/min/1.73 m² and uACR < 30 mg/gCr
(2) Category 2 (moderately increased risk, yellow): eGFR 45–59 mL/min/1.73 m² with uACR < 30 mg/gCr; or eGFR ≥ 60 mL/min/1.73 m² with uACR 30–299 mg/gCr
(3) Category 3 (high risk, orange): eGFR 30–44 mL/min/1.73 m² with uACR < 30 mg/gCr, eGFR 45–59 mL/min/1.73 m² with uACR 30–299 mg/gCr, or eGFR ≥ 60 mL/min/1.73 m² with uACR ≥ 300 mg/gCr
(4) Category 4 (very high-risk, red): eGFR < 30 mL/min/1.73 m² with uACR < 30 mg/gCr, eGFR < 45 mL/min/1.73 m² with uACR 30–299 mg/gCr, or eGFR < 60 mL/min/1.73 m² with uACR ≥ 300 mg/gCr

### Data collection and laboratory measurements

Clinical laboratory parameters and physical examination results were recorded during regular outpatient visits. Clinical history was obtained from electronic medical records. Data extraction was performed in March 2024, with access to medical record numbers restricted to the authors. All data were anonymized prior to analysis.

First morning urine samples were collected from each participant on the day of the visit to minimize variations in hydration status and physical activity [14]. All urine samples were processed using identical procedures: they were aliquoted within 6 hours of collection and stored frozen at −80 °C until analysis.

### Urinary biomarker assays

The uPTM-FetA concentrations were measured using a commercially available enzyme-linked immunosorbent assay (ELISA) kit (Human uPTM3-DKD ELISA, Bio Preventive Medicine Corp., Hsinchu, Taiwan; Lot No.E103R24031801) [27]. Assay precision was confirmed according to the manufacturer’s specifications: the intra-assay and inter-assay coefficients of variation (CV) were 8.02% and 11.09%, respectively. The limit of detection (LOD) was 1.901 ng/mL, and the lower limit of quantification (LOQ) was 5.428 ng/mL. Samples were randomized across plates, and a pooled quality control (QC) sample was included on each plate to monitor batch-to-batch consistency. No sample underwent more than one freeze-thaw cycle prior to analysis. Urinary creatinine was measured using an IDMS-traceable enzymatic method, and uPTM-FetA values were normalized to urinary creatinine (ng/mgCr).

Urinary L-FABP (uL-FABP) concentrations were measured using a commercial ELISA kit (CIMIC Holdings Co., Ltd, Tokyo, Japan) at an external laboratory (SRL Co., Tokyo, Japan). Patients were classified into the high uL-FABP group (>8.4 μg/gCr) or normal uL-FABP group (≤8.4 μg/gCr) based on a previously established cutoff [28].

### Assessment of covariates

Hypertension was defined as either the use of antihypertensive medication or systolic blood pressure ≥140 mmHg and/or diastolic blood pressure ≥90 mmHg at the time of examination. Hyper-low-density lipoprotein (LDL) cholesterolemia was defined as LDL-cholesterol ≥3.62 mmol/L (140 mg/dL) or the use of statins and/or ezetimibe. Hyperuricemia was defined as serum uric acid >416 μmol/L (7.0 mg/dL) or the use of urate-lowering agents (allopurinol or febuxostat)

Diabetic retinopathy was evaluated by an ophthalmologist. Diabetic peripheral neuropathy was diagnosed based on the diagnostic criteria proposed by the Diabetic Neuropathy Study Group in Japan [29], which require the presence of at least two of the following: subjective symptoms such as numbness in both lower extremities, reduced or absent Achilles tendon reflexes, and diminished vibration perception at the medial malleolus. Cerebrovascular disease was diagnosed based on neurological findings at onset and confirmed by brain CT or MRI. Coronary heart disease was identified by a documented history of myocardial infarction or angina pectoris, or diagnosis via coronary angiography. Peripheral artery disease was defined by the presence of ischemic symptoms in the lower limbs and confirmation of arterial stenosis or occlusion by ultrasonography.

### Statistical analysis

Continuous variables are presented as mean ± standard deviation (SD) or median [interquartile range, IQR]. Categorical variables are expressed as numbers (percentages)

Spearman’s rank correlation coefficient was used to assess correlations between continuous variables. Differences between the two groups were analyzed using the Mann-Whitney U test. The proportions of patients with high uPTM-FetA and high uL-FABP levels in each DKD-risk, albuminuria, and eGFR categories were analyzed using the chi-squared (χ^2^) test. To evaluate trends across ordered categories (e.g., DKD-risk, albuminuria, and GFR categories), the Jonckheere–Terpstra test was used for continuous variables (uL-FABP and uPTM-FetA levels), and the Cochran-Armitage trend test was used for categorical proportions (prevalence of high uL-FABP and high uPTM-FetA).

Multivariable logistic regression analyses were conducted to determine the independent association between uPTM-FetA and higher DKD risk (defined as DKD-risk categories 2, 3, and 4 combined). Prior to analysis, the variables were tested for multicollinearity, and the variance inflation factors (VIF) for all variables, including uL-FABP and uPTM-FetA, were <2.0, indicating no significant multicollinearity. Two models were constructed:

(1) Model 1 (full model): adjusted for all potential confounders, including sex, age, duration of diabetes, body mass index, hypertension, renin-angiotensin-aldosterone system (RAAS) inhibitor use, serum albumin, uric acid, and uL-FABP.
(2) Model 2 (primary model): adjusted for sex, age, body mass index, hypertension, renin-angiotensin-aldosterone system (RAAS) inhibitor use, serum albumin, and uric acid. Duration of diabetes and uL-FABP were excluded from this model to minimize potential multicollinearity and to develop a more parsimonious clinical model.

Non-linear Association and Model Validation To assess potential non-linear associations between uPTM-FetA levels and DKD risk, restricted cubic spline (RCS) models were fitted within a multivariable logistic regression framework. Receiver operating characteristic (ROC) analysis was performed to evaluate the discrimination ability of uPTM-FetA. The optimal cutoff value was determined using the Youden index, and sensitivity and specificity were calculated. To estimate the true performance of our primary logistic regression model (Model 2) and check for overfitting, we performed internal validation using a bootstrap procedure with 1,000 resamples. Calibration was assessed by plotting the predicted probabilities against the observed frequencies (calibration plot), and the optimism-corrected area under the curve (AUC) was calculated.

To assess the internal validity of the uPTM-FetA cutoff in our Japanese cohort, we performed a ROC analysis to discriminate between low DKD risk (DKD-risk category 1) and higher DKD risk (DKD-risk categories 2 + 3 + 4) groups. The subjects were classified into high and normal uPTM-FetA groups using the optimal cutoff value derived from the Youden index.

The AUC, sensitivity, and specificity were calculated. The subjects were classified into high and normal uPTM-FetA groups using the optimal cutoff value derived from the Youden index.

This study is exploratory in nature; therefore, statistical significance was defined as a two-sided P-value of <0.05. P-values were not adjusted for multiple comparisons, and results should be interpreted as associations suggestive of future hypotheses. To ensure transparency and reproducibility, all underlying data are provided in S1 Dataset, and the comprehensive statistical workflow is presented in S1 File.

SPSS (version 29.0; IBM Corporation, Armonk, NY, USA) and R (version 4.5.2; R Foundation for Statistical Computing, Vienna, Austria) were used to perform all of the analyses.

### Ethics conduct

Approval of research protocol: This study was conducted by the principles of the 2013 Helsinki Declaration. The Ethics Committee of Edogawa Hospital approved the study protocol (approval number: 2023−31, approval date: August 15, 2023). Informed consent: Eligible patients were enrolled by their attending physicians after obtaining their written informed consent. Registry and the registration no. of the study/trial: The trial was registered in UMIN-CTR under the identifier UMIN000055007.

## Results

### Study population and clinical characteristics

A total of 219 adult patients who met the inclusion criteria were included in the study.

The prevalence of patients across DKD-risk categories was as follows: category 1, n = 103 (47%); category 2, n = 51 (23%); category 3, n = 32 (15%); and category 4, n = 33 (15%).

Table 1 summarizes the clinical characteristics of the study subjects. When comparing the clinical parameters between DKD-risk categories 1 and (combined), several notable differences were observed. Patients in categories 2 + 3 + 4 were older, had a longer diabetes duration, and exhibited higher rates of comorbidities or vascular complications such as hypertension, hyperuricemia, diabetic retinopathy, peripheral neuropathy, and coronary heart disease. The use of RAAS inhibitors and calcium channel blockers was also more frequent in the higher-risk group. Serum uric acid, serum creatinine, uACR, urinary protein-to-creatinine ratio (uPCR), uL-FABP, and uPTM-FetA levels were significantly elevated in higher-risk group whereas serum albumin level was significantly lower.

**Table 1:** Clinical characteristics of the study subjects.

Characteristics	n	All subjects	DKD-risk category		P
			1	2 + 3 + 4	
		(n = 219)	(n = 103)	(n = 116)	
Male (%)	219	58	57	60	0.78
Age (years)	219	66 ± 12	62 ± 12	70 ± 11	<0.01
Duration of diabetes (years)	218	15 ± 10	13 ± 8	17 ± 11	<0.01
Hypertension (%)	219	84	78	90	0.02
Hyper-LDL cholesterolemia (%)	219	78	73	83	0.10
Hyperuricemia (%)	219	25	8	40	<0.01
Diabetic retinopathy (%)	215	34	26	40	0.04
Diabetic peripheral neuropathy (%)	203	35	24	46	<0.01
Cerebrovascular disease (%)	219	11	7	16	0.06
Coronary heart disease (%)	219	16	9	22	<0.01
Insulin use (%)	219	32	28	36	0.25
SGLT2 inhibitor use (%)	219	58	58	59	1.00
Metformin use (%)	219	58	70	47	<0.01
GLP-1 receptor agonist use (%)	219	38	34	42	0.21
DPP-4 inhibitor use (%)	219	37	40	35	0.48
RAAS blocker use (%)	219	65	53	75	<0.01
Calcium channel blocker use (%)	219	49	40	58	0.01
Cholesterol-lowering agent use (%)	219	76	74	78	0.43
Urate-lowering agent use (%)	219	24	8	38	<0.01
Body weight (kg)	218	70.4 ± 16.0	72.2 ± 16.4	68.7 ± 15.5	0.09
Body mass index (kg/m^2^)	218	26.7 ± 4.7	27.1 ± 4.7	26.3 ± 4.6	0.08
Systolic blood pressure (mmHg)	219	137 ± 16	135 ± 16	139 ± 15	0.09
Diastolic blood pressure (mmHg)	219	78 ± 13	79 ± 12	76 ± 13	0.15
Hemoglobin (g/L)	219	140 ± 17	142 ± 16	138 ± 17	0.08
Serum albumin (g/L)	219	43 ± 3	43 ± 3	42 ± 3	<0.01
Total cholesterol (mmol/L)	219	4.32 ± 0.80	4.4 ± 0.7	4.25 ± 0.87	0.06
LDL-cholesterol (mmol/L)	219	2.27 ± 0.61	2.32 ± 0.62	2.22 ± 0.61	0.19
HbA1c (%)	219	7.1 ± 0.8	7.1 ± 0.8	7.1 ± 0.8	0.62
Serum uric acid (μmol/L)	219	290 ± 67	277 ± 66	301 ± 66	0.02
Serum creatinine (μmol/L)	219	81 ± 35	64 ± 12	96 ± 42	<0.01
uACR (/mg/gCr)	219	135.0 ± 354.6	11.7 ± 7.1	244.3 ± 461.2	<0.01
uPCR (g/gCr)	219	0.23 ± 0.53	0.04 ± 0.06	0.39 ± 0.69	<0.01
uL-FABP (μg/gCr)	219	5.81 ± 13.91	1.78 ± 1.15	9.38 ± 18.39	<0.01
Median [IQR]		2.22 [1.26, 3.87]	1.50 [1.06, 2.22]	3.27 [2.03, 7.32]	
uPTM-FetA (ng/mgCr)	219	22.60 ± 30.92	15.53 ± 23.26	28.87 ± 35.33	<0.01
Median [IQR]		12.66 [2.90, 29.20]	7.48 [1.24, 19.49]	18.45 [4.65, 36.05]	

Data are presented as mean ± SD, median [interquartile range], or percentage.

LDL, low-density lipoprotein; SGLT2, sodium-glucose cotransporter 2; GLP-1, glucagon-like peptide-1; DPP-4, dipeptidyl peptidase-4; RAAS, renin-angiotensin-aldosterone system; uACR, urinary albumin-to-creatinine ratio; uPCR, urinary protein-to-creatinine ratio; uL-FABP, urinary liver-type fatty acid binding protein; IQR, interquartile range; uPTM-FetA, urinary post-translationally modified fetuin-A fragments.

### Distribution and correlation of uPTM-FetA

**Fig 1:**
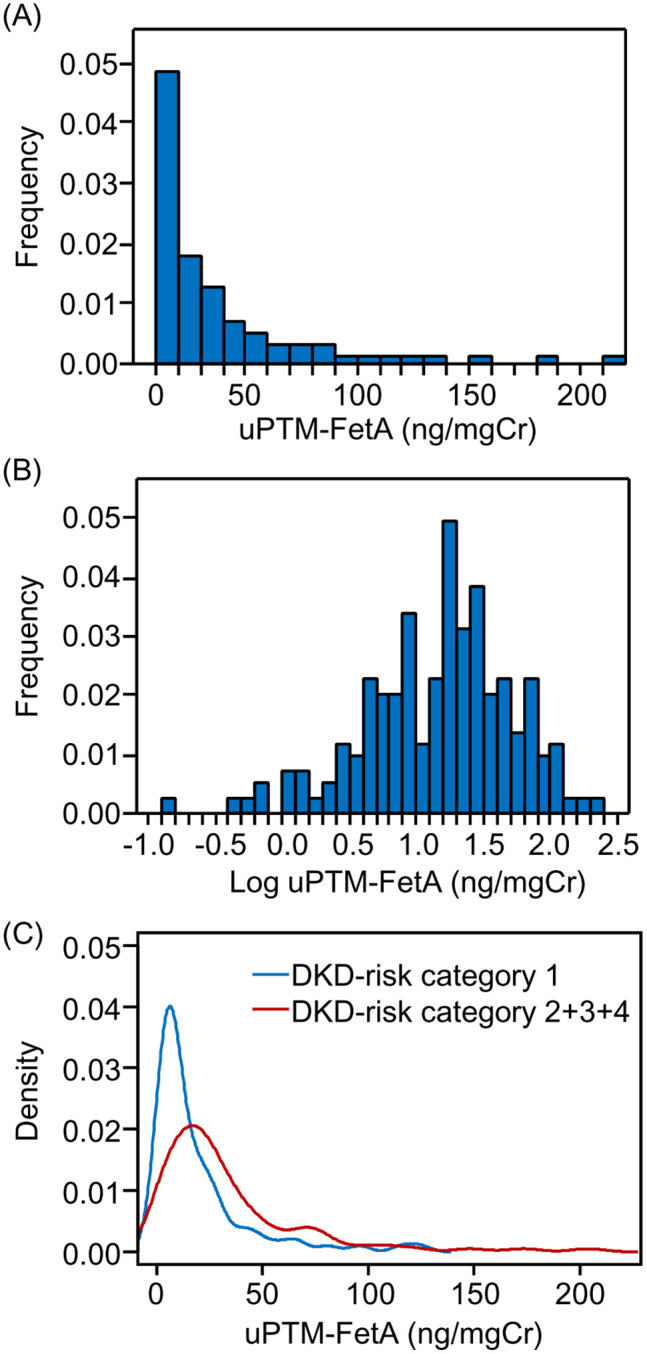
Distribution of urinary PTM-FetA levels. (A) Histogram of raw uPTM-FetA concentrations. (B) Histogram of log-transformed uPTM-FetA concentrations. (C) Density plot of uPTM-FetA levels (raw scale) stratified by DKD risk status (category 1 vs. categories 2 + 3 + 4). Abbreviations: uPTM-FetA, urinary post-translationally modified fetuin-A; DKD, diabetic kidney disease.

**Fig 2:**
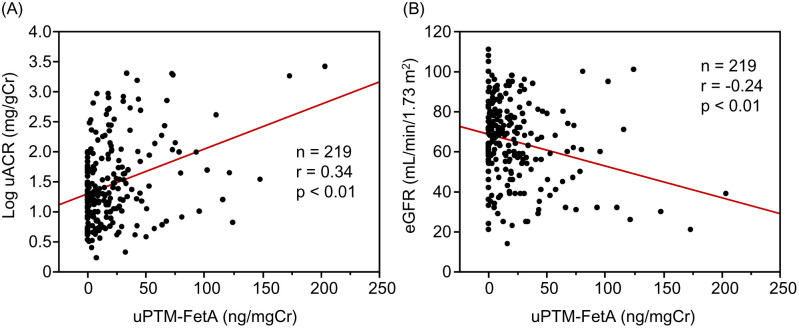
(A) Correlation between uPTM-FetA and existing renal markers. Scatterplots showing the correlation between uPTM-FetA levels and (A) log-transformed uACR and (B) eGFR. Spearman’s rank correlation coefficients (r) and p-values are shown. Abbreviations: uACR, urinary albumin-to-creatinine ratio; eGFR, estimated glomerular filtration rate.

Fig 1A and 1B show the distributions uPTM-FetA concentrations (raw and log-transformed) in the study population. The density plots stratified by risk group (Fig 1C) reveal a progressive rightward shift in uPTM-FetA distribution for the higher-risk categories (2 + 3 + 4), indicating elevated levels compared to the low-risk category. uPTM-FetA levels demonstrated a significant positive correlation with uACR and a significant negative correlation with eGFR (Fig 2). Furthermore, as shown in Table 2, the levels of both uL-FABP and uPTM-FetA significantly increased with advancing DKD-risk categories (P < 0.01 for trend, Jonckheere–Terpstra test).

**Table 2:** uL-FABP and uPTM-FetA stratified by DKD-risk categories.

DKD-risk category	n (%)	uL-FABP (μg/gCr)	uPTM-FetA (ng/mgCr)
1	103 (47.0)	1.78 ± 1.15	15.53 ± 23.26
Median [IQR]		1.50 [1.06, 2.22]	7.48 [1.24, 19.49]
2	51 (23.3)	2.71 ± 1.64	19.10 ± 21.14
Median [IQR]		2.42 [1.57, 3.43]	14.94 [3.50, 26.21]
3	32 (14.6)	4.51 ± 4.23	25.46 ± 23.84
Median [IQR]		3.07 [2.31, 5.26]	18.50 [3.98, 45.17]
4	33 (15.1)	24.42 ± 29.44	47.27 ± 52.18
Median [IQR]		11.9 [5.82, 39.80]	30.64 [10.34, 69.23]
P for trend †		<0.01	<0.01

Data are presented as mean ± SD, median [interquartile range], or percentage.

uL-FABP, urinary liver-type fatty acid binding protein; uPTM-FetA, urinary post-translationally modified fetuin-A fragments, IQR, interquartile range.

† P values for trend were calculated using the Jonckheere-Terpstra test.

### Association between uPTM-FetA and DKD-risk

**Fig 3:**
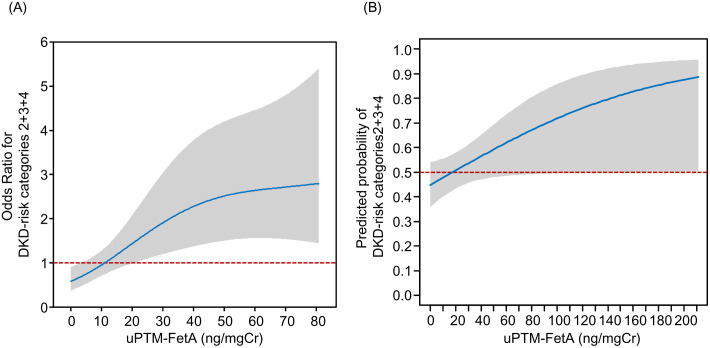
Non-linear association and predictive probabilities of uPTM-FetA for DKD risk. (A) Restricted cubic spline (RCS) analysis of the association between continuous uPTM-FetA levels and the odds ratio (OR) for DKD-risk categories 2–4. Three knots were placed at the 10th, 50th, and 90th percentiles. The solid blue line represents the OR for DKD-risk categories 2 + 3 + 4, with the shaded gray area indicating the 95% confidence interval. The dashed red line indicates an OR of 1.0. (B) Partial effects plot illustrating the predicted probability of being in DKD-risk categories 2 + 3 + 4 across the range of uPTM-FetA levels, adjusted for sex, age, duration of diabetes, body mass index, hypertension, renin-angiotensin-aldosterone system inhibitor use, serum albumin, and serum uric acid. The solid blue line represents the predicted probability for DKD-risk categories 2 + 3 + 4, with the shaded gray area indicating the 95% confidence interval. The dashed red line represents the 50% probability threshold.

RCS analysis demonstrated a significant non-linear association between uPTM-FetA levels and the probability of being in DKD-risk categories 2 + 3 + 4 (P for non-linearity = 0.04), indicating that the odds ratio (OR) for DKD-risk categories 2 + 3 + 4 does not increase at a constant rate across the range of uPTM-FetA (Fig 3A). The OR exceeded 1.0 when uPTM-FetA levels reached approximately 11–12 ng/mgCr, and the lower limit of the 95% confidence interval exceeded 1.0 at approximately 20 ng/mgCr.

To visualize the independent contribution of uPTM-FetA after adjusting for confounders (sex, age, duration of diabetes, body mass index, hypertension, RAAS inhibitor use, serum albumin, and serum uric acid), partial effects plots were generated (Fig 3B). This plot illustrates that the predicted probability of DKD-risk categories 2 + 3 + 4 increases in a non-linear, dose-response manner as uPTM-FetA levels rise, even when other clinical covariates are held constant at their means.

**Fig 4:**
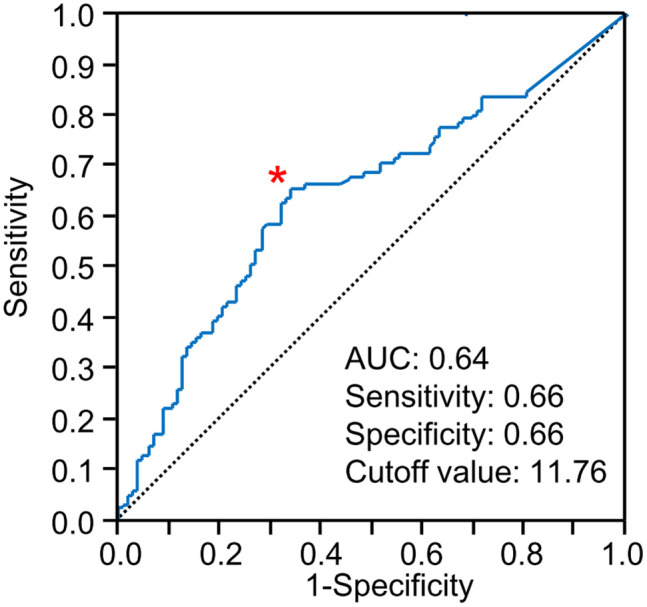
Receiver operating characteristic (ROC) analysis. ROC curve evaluating the diagnostic performance of uPTM-FetA in detecting DKD-risk categories 2 + 3 + 4. The area under the curve (AUC) was 0.64. The optimal data-driven cutoff value was determined to be 11.76 ng/mgCr (Sensitivity: 0.66, Specificity: 0.66) based on the Youden index (indicated by the asterisk).

Fig 4 presents the ROC curve for uPTM-FetA in detecting DKD-risk categories 2 + 3 + 4. The AUC was 0.640. The optimal cutoff value derived from the Youden index was 11.76 ng/mgCr (Sensitivity: 0.66, Specificity: 0.66). Based on this cutoff, the study population was classified into the high uPTM-FetA group (≥11.76 ng/mgCr, n = 111) and the normal uPTM-FetA group (<11.76 ng/mgCr, n = 108). Subsequent comparative analyses were conducted using this threshold.

Table 3 compares the clinical characteristics between these two groups. The high uPTM-FetA group was significantly older, had a longer duration of diabetes, and had a higher prevalence of hypertension and hyperuricemia. Markers of renal injury (uACR, uPCR, uL-FABP) were substantially elevated in the high uPTM-FetA group.

**Table 3:** Comparisons of clinical characteristics between the normal and high uPTM-FetA groups.

Characteristics	Normal	High	P
	(n = 108)	(n = 111)	
Male (%)	62	55	0.29
Age (years)	64 ± 12	69 ± 11.2	<0.01
Duration of diabetes (years)	14 ± 9	16 ± 10	0.03
Hypertension (%)	79	89	0.04
Hyper-LDL cholesterolemia (%)	78	78	0.92
Hyperuricemia (%)	18	32	0.02
Diabetic retinopathy (%)	31	36	0.53
Diabetic peripheral neuropathy (%)	31	39	0.21
Cerebrovascular disease (%)	10	13	0.57
Coronary heart disease (%)	11	20	0.08
Insulin use (%)	26	39	0.04
SGLT2 inhibitor use (%)	65	52	0.06
Metformin use (%)	60	55	0.44
GLP-1 receptor agonist use (%)	36	41	0.50
DPP-4 inhibitor use (%)	40	34	0.39
RAAS blocker use (%)	65	65	0.99
Calcium channel blocker use (%)	46	52	0.38
Cholesterol-lowering agent use (%)	77	76	0.84
Urate-lowering agent use (%)	17	31	0.02
Body weight (kg)	73.7 ± 17.6	67.0 ± 13.5	<0.01
Body mass index (kg/m^2^)	27.3 ± 5.1	26.1 ± 4.2	0.07
Systolic blood pressure (mmHg)	135 ± 15	139 ± 17	0.07
Diastolic blood pressure (mmHg)	78 ± 13	77 ± 13	0.59
Hemoglobin (g/L)	144 ± 16	137 ± 16	<0.01
Serum albumin (g/L)	43 ± 3	42 ± 3	<0.01
Total cholesterol (mmol/L)	4.30 ± 0.69	4.33 ± 0.89	0.92
LDL-cholesterol (mmol/L)	2.29 ± 0.61	2.24 ± 0.62	0.54
HbA1c (%)	7.1 ± 0.8	7.2 ± 0.8	0.37
Serum uric acid (μmol/L)	300 ± 67	280 ± 66	0.04
Serum creatinine (μmol/L)	76 ± 28	86 ± 40	0.11
eGFR (mL/min/1.73m^2^)	68.8 ± 18.7	61.2 ± 20.9	<0.01
uACR (mg/gCr)	46.5 ± 116.4	221.0 ± 470.0	<0.01
uPCR (g/gCr)	0.10 ± 0.21	0.35 ± 0.70	<0.001
uL-FABP (μg/gCr)	3.75 ± 13.20	7.81 ± 14.35	<0.01
Median [IQR]	1.75 [1.16, 2.55]	2.80 [1.59, 6.41]	
uPTM-FetA (ng/mgCr)	3.54 ± 3.61	41.14 ± 34.33	<0.01
Median [IQR]	2.89 [0, 6.57]	28.76 [18.67, 50.18]	

Data are presented as mean ± SD, median [interquartile range], or percentage.

uPTM-FetA, urinary post-translationally modified fetuin-A fragments; LDL, low-density lipoprotein; SGLT2, sodium-glucose cotransporter 2; GLP-1, glucagon-like peptide-1; DPP-4, dipeptidyl peptidase-4; RAAS, renin-angiotensin-aldosterone system; eGFR, estimated glomerular filtration rate; uACR, urinary albumin-to-creatinine ratio; uPCR, urinary protein-to-creatinine ratio; uL-FABP, urinary liver-type fatty acid binding protein; IQR, interquartile range.

### Logistic regression analyses

Table 4 shows the results according to simple logistic regression analyses examining factors associated with DKD-risk categories 2 + 3 + 4. Statistically significant positive associations were identified for age, duration of diabetes, hypertension, hyperuricemia, diabetic retinopathy, peripheral neuropathy, cerebrovascular disease, coronary heart disease, use of RAAS inhibitors or calcium channel blockers, or urate-lowering agents, serum uric acid, creatinine concentrations, uACR, uPCR, uL-FABP, and uPTM-FetA. On the other hand, the use of metformin and serum albumin concentration were inversely associated with the DKD-risk categories 2 + 3 + 4.

**Table 4:** Simple logistic regression analyses of factors for DKD-risk categories 2 + 3 + 4.

Variable	OR [95% CI]	p
Male	1.10 [0.64, 1.88]	0.74
Age (/years)	1.06 [1.03, 1.09]	<0.01
Duration of diabetes (/years)	1.05 [1.02, 1.08]	<0.01
Hypertension	2.49 [1.17, 5.31]	0.02
Hyper-LDL cholesterolemia	1.79 [0.94, 3.43]	0.08
Hyperuricemia	7.80 [3.47, 17.57]	<0.01
Diabetic retinopathy	1.89 [1.06, 3.37]	0.03
Diabetic peripheral neuropathy	2.75 [1.50, 5.03]	<0.01
Cerebrovascular disease	2.52 [1.01, 6.30]	0.048
Coronary heart disease	2.87 [1.27, 6.48]	0.01
Insulin use	1.45 [0.82, 2.57]	0.21
SGLT2 inhibitor use	1.02 [0.59, 1.74]	0.96
Metformin use	0.38 [0.22, 0.66]	<0.01
GLP-1 receptor agonist use	1.42 [0.82, 2.46]	0.21
DPP-4 inhibitor use	0.80 [0.46, 1.38]	0.42
RAAS blocker use	2.62 [1.48, 4.64]	<0.01
Calcium channel blocker use	2.07 [1.21, 3.55]	<0.01
Cholesterol-lowering agents use	1.29 [0.69, 2.41]	0.42
Urate-lowering agents use	7.26 [3.22, 16.37]	<0.01
Body weight (/kg)	0.99 [0.97, 1.00]	0.10
Body mass index (/kg/m^2^)	0.96 [0.91, 1.02]	0.19
Systolic blood pressure (/mmHg)	1.01 [1.00, 1.03]	0.11
Diastolic blood pressure (/mmHg)	0.99 [0.96, 1.01]	0.16
Hemoglobin (/g/L)	0.99 [0.97, 1.00]	0.07
Serum albumin (/g/L)	0.87 [0.80, 0.96]	<0.01
Total cholesterol (/mmol/L)	0.81 [0.58, 1.13]	0.21
LDL-cholesterol (/mmol/L)	0.77 [0.50, 1.20]	0.25
HbA1c (/%)	1.01 [0.73, 1.40]	0.97
Serum uric acid (/μmol/L)	1.01 [1.00, 1.01]	0.01
Serum creatinine (/μmol/L)	1.07 [1.05, 1.09]	<0.01
uACR (/mg/gCr)	1.13 [1.09, 1.17]	<0.01
uPCR (/g/gCr)	>999 [821.2, > 999]	<0.01
uL-FABP (/μg/gCr)	1.87 [1.47, 2.39]	<0.01
High uL-FABP	>10^6^ [13.18, > 999^†^]	0.99
uPTM-FetA (/ng/mgCr)	1.02 [1.01, 1.03]	<0.01
High uPTM-FetA	3.69 [2.13, 6.52]	<0.01

OR, odds ratio; CI, confidence interval; LDL, low-density lipoprotein; SGLT2, sodium-glucose cotransporter 2; GLP-1, glucagon-like peptide-1; DPP-4, dipeptidyl peptidase-4; RAAS, renin-angiotensin-aldosterone system; uACR, urinary albumin-to-creatinine ratio; uPCR, urinary protein-to-creatinine ratio; uL-FABP, urinary liver-type fatty acid binding protein; uPTM-FetA, urinary post-translationally modified fetuin-A fragments. ^†^Not estimable (complete separation).

In the multivariable logistic regression analysis, high uPTM-FetA was significantly associated with DKD-risk categories 2 + 3 + 4 in both Model 1 and Model 2 (Table 5). In Model 2, adjusted for sex, age, hypertension, RAAS inhibitor use, body mass index, serum albumin, and serum uric acid, the high uPTM-FetA group had a significantly higher odds ratio for advanced DKD risk (OR: 3.88, 95% CI: 2.02–7.45, P < 0.01).

**Table 5:** Multiple logistic regression analyses of factors for DKD-Risk categories 2 + 3 + 4.

Variable	Model 1		Model 2	
	OR [95% CI]	P	OR [95% CI]	P
Male	0.92 [0.44, 1.92]	0.82	1.14 [0.59, 2.23]	0.70
Age (/year)	1.05 [1.01, 1.10]	0.02	1.06 [1.02, 1.09]	<0.01
Duration of diabetes (/years)	1.04 [1.00, 1.08]	0.08		
Hypertension	0.60 [0.18, 1.95]	0.39	0.94 [0.32, 2.78]	0.92
RAAS inhibitor use	1.70 [0.66, 4.41]	0.28	1.99 [0.85, 4.68]	0.11
Body mass index (/kg/m^2^)	0.99 [0.92, 1.07]	0.84	0.97 [0.91, 1.05]	0.46
Serum albumin (/g/L)	1.00 [0.88, 1.12]	0.94	0.95 [0.86, 1.06]	0.36
Serum uric acid (/μmol/L)	1.01 [1.00, 1.02]	<0.01	1.01 [1.00, 1.01]	<0.01
High uPTM-FetA	2.52 [1.21, 5.24]	0.01	3.88 [2.02, 7.45]	<0.01
uL-FABP (/μg/gCr)	1.78 [1.36, 2.31]	<0.01		

OR, odds ratio; CI, confidence interval; RAAS, renin-angiotensin-aldosterone system; uPTM-FetA, urinary post-translationally modified fetuin-A fragments; uL-FABP, urinary liver-type fatty acid binding protein.

Sensitivity analyses supported these findings. When uPTM-FetA was analyzed as a standardized continuous variable, each 1-SD increase was associated with a higher risk of DKD (OR: 1.47, P = 0.049, S1 Table). Multinomial logistic regression showed that high uPTM-FetA was consistently associated with each higher risk category (DKD-risk categories 2, 3, and 4) compared to category 1 (S2 Table). In a subgroup analysis limited to early-stage patients (excluding those in albuminuria category A3, or GFR category G4 or G5), the association remained significant (OR: 3.16, P < 0.01, S3 Table). Furthermore, in an additional model adjusting for therapeutic interventions, including sodium-glucose cotransporter 2 (SGLT2) inhibitors, glucagon-like peptide-1 (GLP-1) receptor agonists, calcium channel blockers, and urate-lowering agents, the association between high uPTM-FetA and DKD-risk categories 2 + 3 + 4 remained significant (S4 Table). The direction and magnitude of the odds ratio were consistent with the primary models, confirming the robustness of uPTM-FetA as an independent factor regardless of medication use.

### Model validation

**Fig 5:**
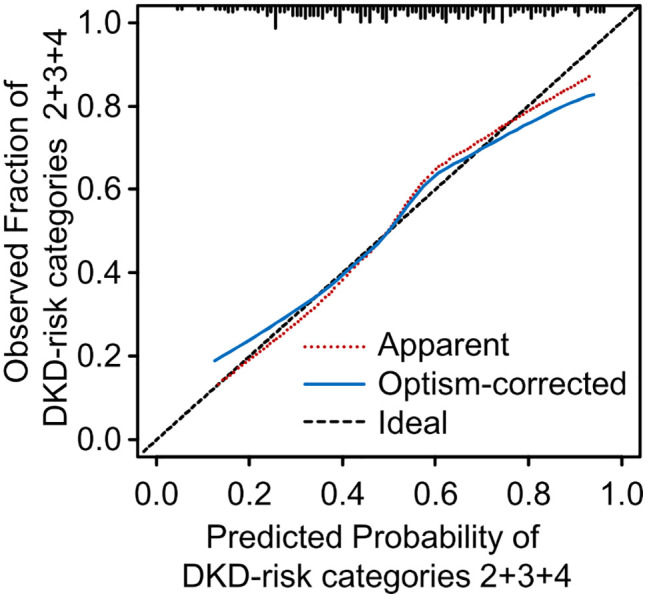
Calibration plot for the multivariable logistic regression model. The plot assesses the agreement between the predicted probabilities (x-axis) and the observed frequencies (y-axis) of DKD risk. The dotted diagonal line represents ideal calibration. The red dashed line represents the apparent performance, while the blue solid line represents the bias-corrected performance, determined through internal validation using a bootstrap procedure with 1,000 resamples.

To assess the potential for overfitting and evaluate the generalizability of Model 2, internal validation was performed using a bootstrap resampling procedure with 1,000 iterations (Fig 5). The apparent AUC of the model was 0.765, while the optimism-corrected AUC was 0.731. The corrected Nagelkerke R^2^ and the calibration slope were 0.193 and 0.815, respectively. These results indicate that Model 2 possesses stable predictive performance and satisfactory calibration, suggesting that the identified association between uPTM-FetA and DKD risk is robust and less likely to be inflated by overfitting to the current dataset.

### Prevalence of elevated biomarkers

**Fig 6:**
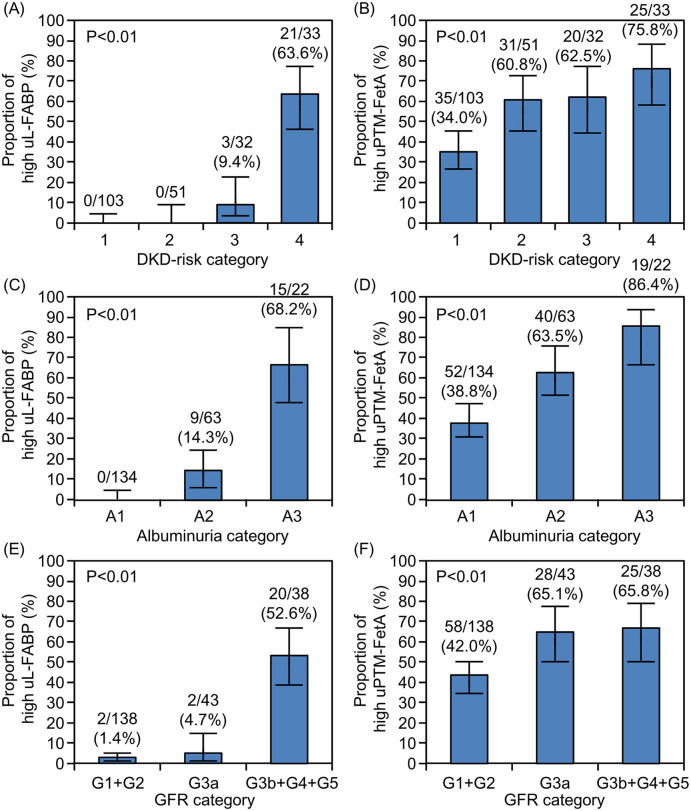
Prevalence of elevated biomarkers stratified by clinical stages. (A) High uL-FABP and (B) high uPTM-FetA (≥11.76 ng/mgCr) across DKD-risk categories (1, 2, 3, and 4). (C) High uL-FABP and (D) high uPTM-FetA stratified by albuminuria stages (A1, A2, and A3). (E) High uL-FABP and (F) high uPTM-FetA stratified by GFR categories (G1 + G2, G3a, and G3b+G4 + G5). Error bars indicate 95% confidence intervals. The numbers above the bars represent the number of cases/ total number of patients in each group (n/N) and the percentage. P-values for trend were assessed using the Cochran-Armitage test. Abbreviations: uL-FABP, urinary liver-type fatty acid-binding protein; uPTM-FetA, urinary post-translationally modified fetuin-A; eGFR, estimated glomerular filtration rate; GFR, glomerular filtration rate.

The prevalence of high uL-FABP significantly increased with the severity of DKD-risk categories (p < 0.01 for trend, Cochran-Armitage trend test). High uL-FABP was observed in 0.0% (95% CI: 0.0–3.5) of patients in category 1 and 0.0% (95% CI: 0.0–7.0) in category 2. In contrast, the frequency of high uL-FABP rose to 9.4% (95% CI: 3.2–24.2) in category 3 and 63.6% (95% CI: 46.6–77.8) in category 4 (Fig 6A). In contrast, high uPTM-FetA was more prevalent across all categories: 34.0% (95% CI: 23.3–46.6) in DKD-risk category 1, rising to 60.8% (95%CI: 47.1–73.0) in category 2, 62.5% (95% CI: 45.3–77.1) in category 3, and 75.8% (95%CI: 59.0–87.2) in category 4 (Fig 6B).

Similar trends were observed when stratified by albuminuria or GFR categories. High uL-FABP was observed in 0.0% (95% CI: 0.0–2.8), 14.3% (95%CI: 7.7–25.0), and 68.2% (95%CI: 47.3–83.6) of patients in albuminuria category A1, A2, and A3, respectively (Fig 6C). In contrast, patients with high uPTM-FetA were more frequent than prevalence of high uL-FABP at 38.8% (95%CI: 31.0–47.3) in the albuminuria category A1, and increased to 63.5% (95% CI: 51.1–74.3), and 86.4% (95% CI: 66.7–95.3) in A2 and A3, respectively (Fig 6D). The prevalence of high uL-FABP was 1.4% (95% CI: 0.3–5.1), 4.7% (95% CI: 1.3–15.5), and 52.6% (95% CI: 37.3–67.5) in the GFR category G1 + G2, G3a, and G3b+G4 + G5, respectively (Fig 6E). In contrast, high uPTM-FetA was more frequent at 42.0% (95 CI: 34.1–50.4) even in GFR category G1 + G2, and increased at 65.1% (95% CI: 50.2–77.6) and 65.8% (95% CI: 49.9–78.8) in G3a and G3b+G4 + G5, respectively (Fig 6F).

## Discussion

### Key findings

This study is the first to investigate the clinical significance of uPTM-FetA in Japanese patients with type 2 diabetes. Our findings revealed that high uPTM-FetA levels are independently associated with higher DKD-risk categories, even after accounting for established markers such as uACR and eGFR. Notably, 38.8% of the patients without albuminuria had high levels of uPTM-FetA. Furthermore, the fact that 42.0% of patients with normal renal function (eGFR ≥ 60 mL/min/1.73 m²) also had high uPTM-FetA levels suggests that this biomarker captures a specific form of renal injury present in the early stages of the disease. While urinary albumin remains the cardinal marker for glomerular damage, our results demonstrate that uPTM-FetA may reflect distinct mechanisms, specifically tubular stress. This suggests that uPTM-FetA serves as a complementary marker to uACR, potentially identifying patients with subclinical renal pathology who are overlooked by current diagnostic criteria.

### Pathophysiological context

In healthy adults, the kidneys typically do not produce fetuin-A [30]. However, when PTECs are injured, they gain the ability to express and release fetuin-A into the tubule lumen [31]. Rudloff et al. demonstrated that PTECs produce local fetuin-A under hypoxic conditions triggered by hypoxia-inducible transcription factors [20]. Fetuin-A in the proximal tubules is thought to protect the kidneys from hypoxia-induced inflammation by inhibiting the conversion of macrophages into pro-inflammatory M1 macrophages and preventing hypoxia-induced fibrosis by opposing transforming growth factor-β signaling [20]. Nangaku emphasized that hypoxia significantly contributes to the progression of kidney disease, potentially occurring at the early stages of renal injury, even before structural damage to the kidneys becomes apparent [10]. Shimizu et al. reported that interstitial fibrosis and tubular atrophy are frequently observed in DKD, even in patients without albuminuria [32]. Based on these findings, elevated uPTM-FetA levels are biologically plausible indicators of tubular damage occurring early in the disease process, prior to overt albuminuria or eGFR decline.

### Comparison with previous studies and cutoff values

Previous longitudinal studies have suggested the predictive value of urinary fetuin-A. Magalhães et al. reported that urinary fetuin-A peptide levels, but not albuminuria, correlated with the eGFR decline slope in 559 patients with type 2 diabetes. Consequently, the authors concluded that urinary fetuin-A peptide levels are a promising biomarker for predicting the progression of DKD earlier than albuminuria [33]. Similarly, Inoue et al. conducted a cross-sectional study of 85 patients with type 2 diabetes and reported that higher urinary fetuin-A excretion was associated with an increased risk of microalbuminuria and reduced kidney function [34]. Recently, Chuang et al. reported that uPTM-FetA predicted progressive eGFR decline in two independent cohorts of different ethnicities (Taiwanese and Dutch), identifying a cutoff value of 7.53 ng/mgCr [25]. In our present study involving Japanese patients, the optimal cutoff value was 11.76 ng/mgCr, which is higher than that reported by Chuang et al [25]. Several factors may account for this discrepancy. First, ethnic differences in baseline biomarker levels or genetic susceptibility to tubular stress may exist between Japanese, Taiwanese, and Dutch populations. Second, differences in urine sampling methods may have contributed; whereas our study strictly utilized first-morning urine samples to minimize variability due to hydration, other studies may have included random spot urine samples. Third, the distribution of disease severity within the cohorts could differ. Nevertheless, the fact that uPTM-FetA showed significant associations with renal risk across diverse populations supports its universal relevance as a DKD biomarker, although population-specific cutoff values may be necessary for clinical application.

### Comparison with uL-FABP

As uL-FABP is a well-established marker of tubular damage, we compared its performance with that of uPTM-FetA. Kamijo et al. previously reported that the uL-FABP level accurately reflects the severity of diabetic nephropathy and serves as a risk factor for disease progression in patients with type 2 diabetes [23]. In a previous study, we found that uL-FABP levels were associated with elevated HbA1c levels and hypertension in patients with type 2 diabetes without albuminuria [35].

In the current study, we observed that uPTM-FetA was already elevated in patients without albuminuria, and in this cross-sectional snapshot, uPTM-FetA was more frequently elevated than uL-FABP in lower KDIGO risk states. This suggests that uPTM-FetA may reflect renal injury through mechanisms distinct from those captured by uL-FABP, and thus serve as a complementary biomarker for risk stratification of DKD in patients with type 2 diabetes. The fact that the association of uPTM-FetA remained significant even in Model 1, adjusted for uL-FABP, is noteworthy. Both uPTM-FetA and uL-FABP reflect tubular injury, but they likely arise from different underlying cellular mechanisms (e.g., hypoxia vs. fatty acid metabolism). By presenting two models, we were able to demonstrate the robustness of uPTM-FetA’s association and its independence from other well-known tubular stress indicators. This suggests that uPTM-FetA captures a distinct component of DKD risk. Furthermore, high uPTM-FetA was independently associated with DKD-risk categories 2 + 3 + 4 in Model 2, even after the inclusion of uL-FABP, reinforcing its independent contribution to risk stratification.

### Strengths and limitations

This study has notable strengths. It provides the first evaluation of uPTM-FetA in a Japanese cohort and utilizes a practical clinic setting to assess the biomarker in a real-world, routine outpatient population. However, we acknowledge several limitations that warrant consideration. First, its cross-sectional design prevents us from drawing conclusions regarding causality, temporal precedence, or prediction of future DKD progression. Therefore, the potential utility of uPTM-FetA for predicting rapid eGFR decline in normoalbuminuric patients requires confirmation in longitudinal studies. Second, this was a single-center study, and the cohort consists of outpatients, which introduces the possibility of selection bias and may limit the generalizability of our findings. Third, while we adjusted for multiple clinical factors, the possibility of residual confounding remains, particularly since therapy intensity (e.g., concomitant use of SGLT2 inhibitors or antihypertensive agents) often serves as a proxy for disease severity itself. Fourth, both uACR and uPTM-FetA were assessed using a single measurement. While the first-morning sample is generally more reliable than random spot urine [14], we recognize that a single measurement is still susceptible to intra-individual variability, which may potentially lead to misclassification of DKD status. Finally, because the sample size in this study was relatively small, further research with a larger cohort is required to establish definitive prognostic cutoffs. The discrepancy in cutoff values compared to non-Japanese cohorts highlights the need for further standardization. Despite these limitations, the robust association observed between uPTM-FetA and DKD risk, independent of uACR and eGFR, suggests its potential utility.

### Future directions

Future, well-designed longitudinal studies are warranted to validate the ability of uPTM-FetA to predict GFR decline and clinical endpoints such as progression to ESKD, independent of traditional biomarkers. Such studies will confirm whether uPTM-FetA can truly serve as a prognostic marker for early DKD progression.

## Conclusion

This study demonstrates that uPTM-FetA is independently associated with higher DKD-risk categories in Japanese patients with type 2 diabetes, even in those without albuminuria or with preserved eGFR. uPTM-FetA appears to identify tubular stress earlier or more sensitively than uL-FABP in this population. While it shows promise as a complementary biomarker to refine risk stratification, its ability to predict future renal function decline needs to be confirmed in prospective cohort studies.

## Supporting information

S1 Dataset Dataset in the present study. All data generated or analyzed during this study are included in supplementary information files. (XLSX)

S1 File Detailed statistical procedures for replication. (PDF)

S1 Table Multiple logistic regression analyses of factors for DKD-risk categories 2 + 3 + 4 using standardized continuous variables (Model 2). (PDF)

S2 Table Multinomial logistic regression analyses using DKD-risk category 1 as the reference group. (PDF)

S3 Table Sensitivity analysis for DKD-risk categories 2 + 3 + 4 in patients with early-stage disease (Model 2). (PDF)

S4 Table Multiple logistic regression analysis for DKD-risk categories 2 + 3 + 4, including the use of SGLT2 inhibitors, GLP-1 receptor agonists, RAAS inhibitors, calcium channel blockers, and urate-lowering agents as independent variables. (PDF)
