# Supplementary material for: Association of urinary post-translationally modified fetuin-A fragments with diabetic kidney disease risk stratification in Japanese patients with type 2 diabetes
Source: PLoS One. 2026 Jul 2;21(7):e0353032. doi: 10.1371/journal.pone.0353032 (PMC13327179; doi:10.1371/journal.pone.0353032)
Supplement: S1 File — (PDF) [file pone.0353032.s002.pdf]

S1 Text. Detailed statistical procedures for replication

1. Software environment

All statistical analyses were performed using **IBM SPSS Statistics Version 29.0** (IBM Corp., Armonk, NY, USA) and **R Version 4.5.2** (The R Foundation for Statistical Computing, Vienna, Austria).

2. Procedures for clinical characteristics and correlations (Tables 1-4, Figs 1 and 2)

1) Descriptive statistics (Table 1):

Mean ± standard deviation (SD) for continuous variables and percentages for categorical variables. Comparisons between DKD-risk categories were performed using the Mann-Whitney U test for continuous variables and the chi-square test for categorical variables.

2) Distribution analysis (Fig 1):

The distribution of uPTM-FetA and its logarithmic transformation were visualized using frequency histograms and density plots.

3) Correlation analysis (Fig 2, Tables 2-4):

Spearman's correlation coefficients were calculated to assess the relationship between uPTM-FetA and renal parameters (uACR, eGFR, and other biomarkers).

3. Logistic regression and trend analysis (Table 5, S1 Tables, S2 Table)

1) Binary logistic regression (Table 5):

Calculated odds ratios (ORs) and 95% confidence intervals (CIs) for DKD-risk categories 2+3+4 vs. DKD-risk category 1.

2) Standardized models (S1 Table):

Continuous variables were standardized using Z-score transformation to report ORs per SD increase.

3) Multinomial logistic regression (S2 Table):

Performed using DKD-risk category 1 as the reference group to evaluate associations with each specific category (2, 3, and 4).

4) Trend analysis:

The linear trend across ordered DKD-risk categories (1 to 4) was assessed using the Jonckheere–Terpstra test.

4. Restricted cubic splines and partial effects (Fig 3)

1) Fig 3A (Splines):

The nonlinear association between uPTM-FetA and DKD-risk categories 2+3+4 was modeled using restricted cubic splines.

2) Fig 3B (Partial effects):

The predicted probability of DKD-risk categories 2+3+4 was plotted by adjusting all other covariates to their mean or reference values using the predict function.

5. Performance and validation (Figs 4 and 5)

1) **ROC analysis (Fig 4):**

Receiver operating characteristic (ROC) curves were generated to calculate the area under the curve (AUC) and determine the optimal cutoff value using the Youden index.

2) Internal validation (Fig 5):

A nonparametric bootstrap resampling procedure (1,000 resamples) was used to generate calibration curves (Apparent vs. Optimism-corrected) to estimate the model's optimism-corrected performance.

6. Proportion analysis (Fig 6)

1) Bar graphs were generated to compare the proportions of subjects with high uL-FABP and high uPTM-FetA across DKD- risk, albuminuria, and GFR categories.

2) Trends in proportions across ordered categories were assessed using the Cochran-Armitage trend test. 95% CIs for proportions were calculated.

7. Sensitivity analysis for early-stage DKD (S3 Table)

1) **Subgroup selection:**

Exclude cases with uACR $\geq$ 300 mg/gCr or eGFR < 30 mL/min/1.73 m^2.

2) Model execution:

Perform multivariable logistic regression using raw clinical units

3) Independent variables:

Include the binary predictor for uPTM-FetA (High $\geq$ 11.76) and covariates.

8. Medication-adjusted sensitivity analysis (S4 Table)

1) Objective:

To verify the robustness of the primary model against potential confounding by therapeutic interventions.

2) Model execution:

A multivariable logistic regression analysis was performed, extending Model 2 to include binary covariates for the use of SGLT2 inhibitors, GLP-1 receptor agonists, RAAS inhibitors, calcium channel blockers, and urate-lowering agents.
