# Supplementary material for: Association of urinary post-translationally modified fetuin-A fragments with diabetic kidney disease risk stratification in Japanese patients with type 2 diabetes
Source: PLoS One. 2026 Jul 2;21(7):e0353032. doi: 10.1371/journal.pone.0353032 (PMC13327179; doi:10.1371/journal.pone.0353032)
Supplement: S1 Table — (PDF) [file pone.0353032.s003.pdf]

S1 Table. Multiple logistic regression analyses of factors for DKD-risk category 2+3+4 using standardized continuous variables (Model 2)

Variable	OR [95% CI]	p
Male	1.15 [0.60, 2.21]	0.67
Age (per 1-SD increase)	1.95 [1.35, 2.88]	<0.01
Hypertension	1.12 [0.39, 3.24]	0.83
RAAS inhibitor use	1.71 [0.76, 3.90]	0.20
Body mass index (per 1-SD increase)	0.89 [0.64, 1.23]	0.47
Serum albumin (per 1-SD increase)	0.79 [0.56, 1.10]	0.17
Serum uric acid (per 1-SD increase)	1.67 [1.17, 2.44]	<0.01
uPTM-FetA (per 1-SD increase)	1.47 [1.03, 2.24]	0.049

OR, odds ratio; CI, confidence interval; SD, standard deviation; RAAS, renin-angiotensin-aldosterone system; uPTM-FetA, urinary post-translationally modified fetuin-A fragment
