# Supplementary material for: Association of urinary post-translationally modified fetuin-A fragments with diabetic kidney disease risk stratification in Japanese patients with type 2 diabetes
Source: PLoS One. 2026 Jul 2;21(7):e0353032. doi: 10.1371/journal.pone.0353032 (PMC13327179; doi:10.1371/journal.pone.0353032)
Supplement: S2 Table — (PDF) [file pone.0353032.s004.pdf]

S2 Table. Multinomial logistic regression analyses using DKD-risk category 1 as the reference group

Variable	Model 1		Model 2	
	OR [95% CI]	P	OR [95% CI]	P
vs. DKD-risk category 2				
Male	1.29 [0.53, 3.19]	0.57	1.41 [0.62, 3.32]	0.42
Age (/year)	1.02 [0.98, 1.07]	0.35	1.03 [0.99, 1.07]	0.21
Duration of diabetes (/years)	1.03 [0.98, 1.08]	0.30		
Body mass index (/kg/m^2)	1.01 [0.92, 1.10]	0.84	1.00 [0.92, 1.09]	1.00
Hypertension	0.62 [0.17, 2.27]	0.47	0.70 [0.19, 2.45]	0.57
RAAS inhibitor use	1.63 [0.56, 4.95]	0.38	2.12 [0.78, 6.23]	0.16
Serum albumin (/g/L)	1.16 [0.31, 4.33]	0.83	0.83 [0.23, 2.91]	0.77
Serum uric acid (/μmol/L)	1.21 [0.78, 1.88]	0.39	1.09 [0.73, 1.64]	0.66
High uPTM-FetA	3.01 [1.35, 6.91]	<0.01	3.60 [1.67, 8.07]	<0.01
uL-FABP (/μg/gCr)	1.56 [1.17, 2.16]	<0.01		
vs. DKD-risk category 3				
Male	1.14 [0.34, 4.06]	0.84	0.84 [0.31, 2.33]	0.74

Age (/year)	1.15 [1.07, 1.27]	<0.01	1.11 [1.05, 1.18]	<0.01
Duration of diabetes (/years)	1.05 [0.98, 1.13]	0.21		
Body mass index (/kg/m^2)	1.00 [0.86, 1.17]	0.99	0.97 [0.86, 1.10]	0.66
Hypertension	0.51 [0.05, 6.46]	0.57	0.72 [0.11, 5.12]	0.73
RAAS inhibitor use	1.36 [0.29, 6.76]	0.70	1.66 [0.42, 6.83]	0.47
Serum albumin (/g/L)	2.26 [0.26, 16.85]	0.42	0.48 [0.08, 2.49]	0.40
Serum uric acid (/µmol/L)	2.85 [1.50, 6.01]	<0.01	2.26 [1.31, 4.13]	<0.01
High uPTM-FetA	3.03 [0.91, 10.84]	0.08	4.19[1.50, 12.62]	<0.01
uL-FABP (/µg/gCr)	2.30[1.55, 3.70]	<0.01		
vs. DKD-risk category 4				
Male	0.21 [0.01, 2.85]	0.27	1.63 [0.46, 6.08]	0.45
Age (/year)	1.24 [1.06, 1.56]	0.02	1.09 [1.02, 1.18]	0.03
Duration of diabetes (/years)	1.05 [0.94, 1.17]	0.41		
Body mass index (/kg/m^2)	0.98 [0.74, 1.26]	0.84	1.00 [0.88, 1.13]	0.99
Hypertension	0.02 [0.00, 2.24]	0.13	0.72 [0.06, 18.33]	0.81
RAAS inhibitor use	190.89 [1.69, >999]	0.09	5.94 [1.16, 40.29]	0.05

Serum albumin (/g/L)	14.39 [0.38, >999]	0.17	1.04 [0.16, 6.63]	0.97
Serum uric acid (/μmol/L)	10.09 [2.60, 90.79]	<0.01	3.47 [1.88, 7.21]	<0.01
High uPTM-FetA	0.81 [0.06, 9.23]	0.87	10.2 [2.94, 43.54]	<0.01
uL-FABP (/μg/gCr)	3.89 [2.04, 12.61]	<0.01		

OR, odds ratio; CI, confidence interval; RAAS, renin-angiotensin-aldosterone system; uPTM-FetA, urinary post-translationally modified fetuin-A fragments; uL-FABP, urinary liver-type fatty acid binding protein.
