# Supplementary material for: Association of urinary post-translationally modified fetuin-A fragments with diabetic kidney disease risk stratification in Japanese patients with type 2 diabetes
Source: PLoS One. 2026 Jul 2;21(7):e0353032. doi: 10.1371/journal.pone.0353032 (PMC13327179; doi:10.1371/journal.pone.0353032)
Supplement: S3 Table — (PDF) [file pone.0353032.s005.pdf]

S3 Table. Sensitivity analysis for DKD-risk categories 2+3+4 in patients with early-stage disease (Model 2)

	OR [95% CI]	p
Male	1.01 [0.51, 2.03]	0.97
Age (/year)	1.06 [1.03, 1.10]	<0.01
Hypertension	0.76 [0.25, 2.30]	0.62
RAAS inhibitor use	1.94 [0.81, 4.78]	0.14
Body mass index (/kg/m^2)	0.98 [0.90, 1.05]	0.53
Serum albumin (/g/L)	0.97 [0.86, 1.08]	0.55
Serum uric acid (/µmol/L)	1.01 [1.00, 1.01]	0.02
High uPTM-FetA	3.16 [1.61, 6.39]	<0.01

OR, odds ratio; CI, confidence interval; RAAS, renin-angiotensin-aldosterone system; uPTM-FetA, urinary post-translationally modified fetuin-A fragments
