# Supplementary material for: Association of urinary post-translationally modified fetuin-A fragments with diabetic kidney disease risk stratification in Japanese patients with type 2 diabetes
Source: PLoS One. 2026 Jul 2;21(7):e0353032. doi: 10.1371/journal.pone.0353032 (PMC13327179; doi:10.1371/journal.pone.0353032)
Supplement: S4 Table — (PDF) [file pone.0353032.s006.pdf]

S4 Table. Multiple logistic regression analysis for DKD-risk categories 2+3+4, including the use of SGLT2 inhibitors, GLP-1 receptor agonists, RAAS inhibitors, calcium channel blockers, and urate-lowering agents as independent variables.

Variable	Model 1		Model 2	
	Odds Ratio [95% CI]	p	Odds Ratio [95% CI]	p
Male	0.65 [0.29, 1.47]	0.30	0.74 [0.35, 1.55]	0.42
Age (/year)	1.07 [1.01, 1.12]	0.01	1.07 [1.03, 1.12]	<0.01
Duration of diabetes (/years)	1.03 [0.98, 1.08]	0.21		
Hypertension	0.37 [0.10, 1.38]	0.14	0.52 [0.15, 1.77]	0.30
SGLT2 inhibitor use	1.79 [0.75, 4.25]	0.19	2.04 [0.93, 4.47]	0.08
GLP-1 receptor agonist use	2.49 [1.04, 5.95]	0.04	3.13 [1.39, 7.04]	<0.01
RAAS inhibitor use	1.48 [0.54, 4.04]	0.44	1.62 [0.65, 4.04]	0.30
Calcium channel blocker use	1.45 [0.64, 3.27]	0.37	1.56 [0.74, 3.28]	0.24
Urate-lowering agents use	3.97 [1.36, 11.70]	0.01	5.73 [2.15, 15.30]	<0.01
Body mass index (/kg/m^2)	0.95 [0.87, 1.04]	0.30	0.92 [0.84, 1.00]	0.048
Serum albumin (/g/L)	1.00 [0.88, 1.14]	0.97	0.95 [0.84, 1.07]	0.37
Serum uric acid (/µmol/L)	1.01 [1.00, 1.02]	<0.01	1.01 [1.00, 1.02]	<0.01
High uPTM-FetA	2.57 [1.18, 5.58]	0.02	3.27 [1.61, 6.64]	<0.01

uL-FABP (/μg/gCr)	1.67 [1.27, 2.19]	<0.01

OR, odds ratio; CI, confidence interval; SGLT2, sodium-glucose cotransporter 2; GLP-1, glucagon-like peptide-1; RAAS, renin-angiotensin-aldosterone system; uL-FABP, urinary liver-type fatty acid binding protein; uPTM-FetA, urinary post-translationally modified fetuin-A fragments.
