# Supplementary material for: Association of urinary post-translationally modified fetuin-A fragments with diabetic kidney disease risk stratification in Japanese patients with type 2 diabetes
Source: PLoS One. 2026 Jul 2;21(7):e0353032. doi: 10.1371/journal.pone.0353032 (PMC13327179; doi:10.1371/journal.pone.0353032)
Supplement: Attachment — Submitted filename: Response to Reviewers.docx [file pone.0353032.s007.docx]

**Responses to Academic Editor and Reviewers**

**Journal Requirements**

**Requirement 1 (Style requirements):** Please ensure that your manuscript meets PLOS ONE's style requirements, including those for file naming.

**Response:** We have thoroughly reviewed the PLOS ONE style templates and formatted both the manuscript and file names accordingly.

**Requirement 2 (Competing Interests):** We note that you received funding from a commercial source: “Novo Nordisk Pharma Ltd”. Please provide an amended Competing Interests Statement...

**Response:** We would like to clarify that we did not receive any research funding or study support from Novo Nordisk Pharma Ltd. Our relationship with Novo Nordisk Pharma Ltd. is limited to the receipt of lecture fees. We believe that the reference to “funding from a commercial source” may have resulted from a misunderstanding of our initial disclosure. We would appreciate your consideration of the following amended Competing Interests Statement:

Hiroyuki Ito received lecture fees from Novo Nordisk Pharma Ltd., Eli Lilly Japan KK, Sanofi KK, Astellas Pharma, Kowa Company, Ltd., Taisho Pharmaceutical Co., Ltd., Sumitomo Pharma Co., Ltd., Boehringer Ingelheim, Daiichi Sankyo Company, Novartis Pharma KK, Takeda Pharmaceutical Company Ltd., MSD KK, Terumo Corporation, Mochida Pharmaceuticals, Teijin Pharma, Kissei Pharmaceuticals, Mitsubishi Tanabe Pharma Corporation, Sanwa Kagaku Kenkyusho, AstraZeneca KK, Kyowa Kirin Co. Ltd., Otsuka Pharmaceutical Co., Ltd., Bayer Yakuhin, Ltd., EA Pharma Co., Ltd., Ono Pharmaceutical Co., Ltd., and Viatris Inc., and received consulting fees from Becton, Dickinson and Company.

Suzuko Matsumoto received lecture fees from Eli Lilly Japan KK, Novo Nordisk Pharma Ltd., Astellas Pharma, Kyowa Kirin Co., Ltd., and AstraZeneca KK.

Hideyuki Inoue received lecture fees from Novartis Pharma KK, AstraZeneca KK, and Mochida Pharmaceuticals.

Shinichi Antoku received lecture fees from Kyowa Kirin Co., Ltd., Sanofi KK, Taisho Pharmaceutical Co., Ltd., Daiichi Sankyo Company, Novo Nordisk Pharma Ltd., Novartis Pharma KK, AstraZeneca KK, and Otsuka Pharmaceutical Co., Ltd.

Toshiko Mori received lecture fees from Novartis Pharma KK.

Chizuko Yukawa has no conflict of interest.

This does not alter our adherence to PLOS ONE policies on sharing data and materials.

**Requirement 3 (Data Anonymization):** We note that there is identifying data in the Supporting Information file < Supplement 1 (Deta Set).xlsx>... Please remove or anonymize all personal information.

**Response:** In accordance with this requirement, we have removed all potentially identifying information from the **S1 Dataset** to ensure participant privacy. The fully anonymized dataset has been re-uploaded as part of this revision.

**Requirement 4 (Citing references):** If the reviewer comments include a recommendation to cite specific previously published works, please review and evaluate these publications to determine whether they are relevant and should be cited. There is no requirement to cite these works unless the editor has indicated otherwise.

**Response:** Thank you for this guidance. While no specific publications were recommended by the reviewers, we have thoroughly reviewed recent literature pertaining to uPTM-FetA, DKD biomarkers, and tubular injury markers. Consequently, we have incorporated several relevant studies into the revised manuscript to better contextualize our findings within the current landscape of diabetic kidney disease research. We believe these additional citations have strengthened the theoretical framework and clinical relevance of our study.

**Response to Academic Editor and Reviewers**

**Academic Editor Comments**

**Major comments**

**Comment 1 (Cross-sectional design and causal language):** The manuscript occasionally implies early detection/predictive potential. Given the design, please avoid inference about prediction or temporal precedence and reframe conclusions strictly as associations. Consider moving predictive language to “Future directions” and explicitly add a STROBE-consistent “Limitations” paragraph.

**Response:** We agree with the Editor’s assessment. Given the cross-sectional nature of this study, we have carefully revised the manuscript to avoid language implying causality or predictive capacity (e.g., replacing "predict" with "is associated with"). As suggested, speculative discussion regarding the predictive potential of uPTM-FetA has been moved to the "Future directions" section. Furthermore, we have added a dedicated "Limitations" subsection within the Discussion to explicitly address the inherent constraints of our study design, ensuring full compliance with STROBE guidelines.

**Comment 2 (Choice and transportability of the uPTM-FetA cutoff (7.53 ng/mgCr)):** The dichotomization threshold is imported from external cohorts. Provide a justification for transportability to this Japanese clinic population: distribution plots, within-sample ROC analysis vs KDIGO categories, and sensitivity analyses using uPTM-FetA as (a) a continuous predictor (with restricted cubic splines) and (b) alternative data-driven thresholds. Right now, heavy reliance on a single external cut-point risks misclassification and loss of information.

**Response:** We agree that a robust justification for the threshold is essential for the transportability of the biomarker to our specific study population. Accordingly, we have performed the following additional analyses:

1. Distribution plots: To visualize the data structure, we have included histograms of uPTM-FetA levels (both raw and log-transformed) in the revised manuscript (**Fig 1A and 1B**). Additionally, a density plot stratified by DKD-risk category has been added to show the shift in uPTM-FetA distribution (**Fig 1C**).

2. Within-sample ROC Analysis: We conducted a ROC analysis to evaluate the diagnostic performance of uPTM-FetA in identifying DKD-risk categories 2+3+4. The area under the curve (AUC) was 0.64. Based on the Youden index, the optimal data-driven cutoff for this cohort was identified as 11.76 ng/mgCr (Sensitivity: 0.66, Specificity: 0.66; **Fig 4**). Consequently, we have adopted this specific cutoff value (11.76 ng/mgCr) for our binary analyses in this study. The discrepancy between this data-driven threshold and previously reported values (e.g., 7.53 ng/mgCr) is now addressed in the Discussion section.

3. Continuous variable analysis (Restricted cubic splines): To avoid potential information loss from dichotomization, we performed sensitivity analyses using uPTM-FetA as a continuous variable. The restricted cubic spline (RCS) model confirmed a significant non-linear association between uPTM-FetA levels and the increased odds of higher DKD-risk categories, reinforcing the robustness of our findings (**Fig 3A**).

**Comment 3 (Model specification and potential mediator/collinearity handling):** You present two multivariable models: one with uL-FABP and one excluding it, after noting VIF<2 for all variables. Please clarify the a priori causal framework: Is uL-FABP a competing biomarker, a mediator, or simply a correlated marker of tubular injury? If it’s a mediator, Model 1 may be “over-adjusted”; if not, exclusion in Model 2 risks omitted-variable bias. Pre-register or at least justify this strategy and report both sets fully (including calibrated probabilities and overall fit). Consider providing a DAG to clarify adjustment choices.

**Response:** We appreciate this insightful request to clarify our conceptual framework. We consider uL-FABP and uPTM-FetA to be correlated markers reflecting distinct, though potentially overlapping, pathological pathways of tubular injury. We do not hypothesize that uL-FABP acts as a direct mediator in the causal pathway between uPTM-FetA and DKD severity.

To address the risk of over-adjustment while ensuring robust results, we have adopted the following strategy:

1. Primary model (Model 2): We designate Model 2 (unadjusted for uL-FABP) as our primary model to evaluate the independent association of uPTM-FetA with DKD risk categories.

2. Sensitivity analysis (Model 1): Model 1 (adjusted for uL-FABP) is presented as a sensitivity analysis. This model confirms that the association between uPTM-FetA and DKD-risk remains statistically significant even after accounting for uL-FABP, a well-established marker of tubular stress. This demonstrates that uPTM-FetA provides distinct clinical information independent of uL-FABP.

We have added a statement to the Methods section to clarify this a priori modeling framework. Furthermore, the physiological and clinical differences between uPTM-FetA and uL-FABP are now elaborated upon in the Discussion section. We believe this strategy effectively addresses the concern of omitted-variable bias while avoiding the pitfalls of over-adjustment.

**Comment 4 (Outcome definition and risk-category amalgamation):** The primary outcome collapses KDIGO categories 2–4 vs 1. Because these categories integrate both albuminuria and eGFR (with multiple pathways to “high risk”), the biology differs across 2, 3, and 4. Please (i) report separate models for each category vs 1, (ii) add trend tests across ordered risk categories, and (iii) provide partial effects plots for interpretability.

**Response:** We acknowledge that biological pathophysiology may differ across KDIGO risk categories 2, 3, and 4. As suggested, we have addressed this by performing more granular analyses to evaluate the association of uPTM-FetA across the full spectrum of DKD risk:

1. Multinomial logistic regression: To evaluate each category separately, we performed multinomial logistic regression using KDIGO category 1 as the reference group. The odds ratios (ORs) for uPTM-FetA exposure for categories 2, 3, and 4 are now reported individually in **S2 Table**.

2. Trend test: To assess the dose-response relationship, we conducted the Jonckheere–Terpstra test for linear trends across the ordered KDIGO risk categories (1 to 4). This analysis confirmed a highly significant progressive increase in uPTM-FetA levels associated with advancing risk stages (p for trend < 0.01).

3. Partial effects plots: To enhance interpretability, we have included a partial effects plot (**Fig 3B**). This figure illustrates the predicted probabilities of DKD-risk (categories 2+3+4) across the range of uPTM-FetA values, after adjusting for potential confounders.

**Comment 5 (Confounding by medication** **and disease severity):** Several drug classes (e.g., SGLT2i, RAAS blockade, urate-lowering agents) and comorbidities differ across groups and associate with DKD severity (see Tables; many differences are reported). At minimum, adjust your primary model for classes plausibly linked to tubular biomarkers and DKD severity, provide a medication-adjusted sensitivity analysis, and consider propensity-score or inverse probability weighting as robustness checks.

**Response:** We agree that medications affecting renal hemodynamics or DKD progression could act as potential confounders. To address this, we performed a medication-adjusted sensitivity analysis as requested.

We constructed an extended multivariable model that includes the use of SGLT2 inhibitors, GLP-1 receptor agonists, RAAS inhibitors, calcium channel blockers, and urate-lowering agents as additional independent variables (**S4 Table**). The results demonstrated that even after rigorous adjustment for these therapeutic classes, there were no significant changes in the direction, magnitude, or statistical significance of the odds ratio for high uPTM-FetA. These findings confirm the independent association of uPTM-FetA with DKD risk and underline the robustness of our primary model against potential confounding by medication use.

**Comment 6 (Measurement and pre-analytical detail for the ELISA):** Add essential assay characteristics: lot numbers, intra/inter-assay CVs, LOD/LOQ, sample handling (first-morning vs random spot is stated; please add storage temp, freeze-thaw cycles), creatinine assays used for normalization, and whether batch effects were controlled (e.g., randomization of plates, inclusion of pooled QC). These are critical for biomarker papers and currently under-described.

**Response:** We fully recognize the importance of providing detailed assay characteristics for the transparency and reliability of biomarker studies. In response to this comment, we have extensively updated the Methods section to include the following technical specifications:

1. Assay performance: We have specified the lot numbers of the ELISA kits used, the intra- and inter-assay coefficients of variation (CVs), and the limit of detection (LOD) and limit of quantification (LOQ).

2. Sample handling and storage: We have clarified that spot urine samples were stored at -80°C immediately after collection and processing. We also explicitly stated that samples underwent no more than one freeze-thaw cycle prior to analysis.

3. Creatinine normalization: Details regarding the enzymatic assay used for urinary creatinine measurement and its traceability have been added.

4. Quality and batch control: To minimize batch effects, samples were randomized across plates, and internal pooled quality control (QC) samples were included in each run to monitor assay stability.

These additions ensure that our methodology adheres to the rigorous standards required for biomarker validation.

**Comment 7 (Internal validity: multiple testing and model stability):** Numerous univariate comparisons are presented. Please specify how you addressed multiplicity (e.g., false discovery rate) or emphasize that p-values are exploratory. For the logistic models, provide optimism-corrected performance (bootstrap) and calibration (e.g., calibration slope/intercept), even though the outcome is a categorical risk state.

**Response:** We have addressed the concerns regarding internal validity and model stability through the following two key revisions:

1. Multiplicity: We have explicitly stated in the Statistical Analysis section that this is an exploratory study. Consequently, p-values were not adjusted for multiple comparisons, and our findings should be interpreted as suggestive of associations intended to generate hypotheses for future research.

2. Model stability and internal validation: To address the potential for overfitting and to evaluate the internal validity of Model 2, we performed a rigorous internal validation using a nonparametric bootstrap resampling procedure (1,000 iterations).

- Discriminative ability: While the apparent AUC was 0.765, the optimism-corrected AUC was 0.731, indicating satisfactory discriminative performance even after accounting for potential overfitting.

- Calibration: The calibration slope was 0.815 (approaching the ideal value of 1.0), and the corrected Nagelkerke R² was 0.193.

- Visualization: To visually supplement these findings, we have generated a calibration plot (**Fig 5**), which demonstrates excellent agreement between the predicted and observed probabilities of DKD-risk categories across the entire spectrum.

2. Model stability: To address the potential for overfitting and to evaluate the internal validity of Model 2, we performed an internal validation using a bootstrap resampling procedure (1,000 iterations). The validation results demonstrated the stability and reliability of the model. While the apparent AUC was 0.765, the optimism-corrected AUC was 0.731, indicating a satisfactory discriminative ability even after adjusting for potential overfitting. Furthermore, the calibration slope was 0.815, which is close to the ideal value of 1.0, and the corrected Nagelkerke R^2^ was 0.193. To visually supplement these findings, we have also generated a calibration plot (**Fig 5**), which shows an excellent agreement between the predicted and observed probabilities across the entire risk spectrum.

These validation results confirm that the association between high uPTM-FetA and DKD risk is robust and that our model maintains stable performance for clinical risk assessment. These details have been incorporated into the Methods and Results sections.

**Comment 8 (Interpretation versus uL-FABP):** The statement that uPTM-FetA “rises earlier than uL-FABP” is inferred from cross-sectional proportions (e.g., no high uL-FABP in lower DKD risk groups) rather than longitudinal change. Please temper this claim and reframe as “uPTM-FetA was more frequently elevated than uL-FABP in lower KDIGO risk states in this cross-sectional snapshot,” or demonstrate incremental discrimination (e.g., likelihood-ratio test for nested models, partial AUC) within this design.

**Response:** We apologize for the overstatement regarding the temporal relationship between these biomarkers. We recognize that our cross-sectional study design does not allow for definitive conclusions about the timing of biomarker elevation. As suggested, we have carefully revised the Abstract and Discussion sections to replace any language implying temporal precedence with the recommended phrasing: "uPTM-FetA was more frequently elevated than uL-FABP in lower KDIGO risk states in this cross-sectional snapshot." These revisions ensure that our conclusions are strictly aligned with the observational nature of the data.

**Comment 9 (COI/Data availability inconsistencies):** The submission packet contains both a templated “NO authors have competing interests” statement and, elsewhere, detailed financial relationships; this must be reconciled to PLOS standards. Similarly, the Data Availability statement asserts that “All relevant data are within the manuscript and its Supporting Information files” — ensure the S1 dataset actually contains row-level, de-identified data and a codebook that reproduce all tables/figures.

**Response:** We have reconciled these inconsistencies to ensure full compliance with PLOS ONE standards:

1. Competing interests: We have updated the Competing Interests section to provide a single, comprehensive statement that includes all relevant financial disclosures for all authors.

2. Data availability: We have confirmed that the **S1 Dataset** contains row-level, fully de-identified data. To ensure full transparency and facilitate the independent replication of our findings, we have added **S1 Text** (Detailed statistical procedures for replication). This comprehensive document provides a step-by-step description of our statistical workflow.

**Specific/Section-by-Section Notes**

**Title/Abstract:**

• Clear scope. In the Conclusions, remove predictive framing (“early detection”) or qualify it as hypothesis-generating given the design.

• Include the study dates and setting in the abstract Methods for clarity (Edogawa Hospital; 2023-11-16 to 2024-02-26) to align with STROBE.

**Response:** We sincerely appreciate the reviewer’s constructive feedback regarding the framing of our conclusions. We agree that the cross-sectional design of this study precludes definitive claims regarding "early detection" or the "prediction" of future disease progression.

Accordingly, we have implemented the following revisions to the Abstract:

1. Reframing conclusions: We have removed all predictive terminology from the Conclusion section. The revised text now emphasizes the association between uPTM-FetA and current DKD risk categories, explicitly qualifying these findings as hypothesis-generating for future longitudinal studies.

2. Study setting and period: In accordance with STROBE guidelines, we have updated the Methods section of the Abstract to include the study setting (Edogawa Hospital) and the specific data collection period (November 16, 2023, to February 26, 2024).

**Introduction:**

• Nicely motivated. Consider briefly differentiating mechanistic pathways (glomerular vs tubular injury) to frame why uPTM-FetA might capture injury distinct from albuminuria and uL-FABP, and cite the KDIGO risk grid’s composite nature to justify stratified analyses.

**Response:** We appreciate this constructive suggestion to clarify the physiological rationale of our study. In the revised Introduction, we have added a concise description differentiating the mechanistic pathways of glomerular injury (represented by albuminuria) from those of tubular injury (captured by markers such as uPTM-FetA and uL-FABP).

Specifically, we framed uPTM-FetA as a marker that may reflect distinct aspects of tubular stress or damage that are not fully captured by glomerular markers. Furthermore, we now explicitly cite the composite nature of the KDIGO risk grid, which integrates both eGFR and albuminuria, to justify our use of stratified analyses across combined risk categories. This addition provides a stronger theoretical foundation for assessing uPTM-FetA’s clinical utility within the existing diagnostic framework.

**Methods:**

**Response:**

• KDIGO risk categories and biomarker cut-points are well defined. Please correct small typos (“Mesurements”), and add analytic decisions: handling of outliers, missingness, transformations (log-uPTM-FetA, log-uACR), and model diagnostics.

**Response:** We have corrected the typographical error “Mesurements” to “Measurements” throughout the manuscript. Regarding our analytic decisions, we have added the following details to the Statistical Analysis section:

1. Missing data: As the proportion of missing values for most covariates was negligible (see **Table 1**), we performed a complete case analysis without imputation.

2. Handling of outliers: Outliers were identified and verified; however, they were retained in the final models after sensitivity analyses confirmed that their exclusion did not significantly alter the direction or statistical significance of the results.

3. Data transformations: We initially applied natural logarithmic transformations to uPTM-FetA, uL-FABP, and uACR to reduce skewness. However, as these variables did not strictly follow a normal distribution even after transformation (as confirmed by the Shapiro-Wilk test), we adopted the following robust statistical approach:

- Non-parametric tests: For univariate comparisons between groups, we used the Mann-Whitney U test, which do not assume a normal distribution.

- Visualization: We have provided histograms and density plots (**Fig 1**) to remain transparent about the actual data distribution, allowing for a clear assessment of the skewness.

4. Model Diagnostics: We have included a description of model diagnostics, including the assessment of multicollinearity (VIF <2) and the evaluation of model calibration as detailed in our response to Comment 7.
• Ethics and registration are appropriately documented (committee, number/date, UMIN ID) — good
• Explicitly state the eGFR formula (Japanese Society of Nephrology) and lab methods; you currently reference the formula but not its equation or creatinine assay traceability

**Response:** In accordance with this recommendation, we have updated the Methods section to explicitly state the Japanese Society of Nephrology (JSN) equation used for eGFR calculation:

eGFR (mL/min/1.73 m^2^) = 194 x Cr^-1.094^ x Age^-0.287^ (x 0.739 if female)

Furthermore, we have provided details regarding the enzymatic method used for serum creatinine measurement and confirmed its traceability to international standard reference materials.

**Results**
• Provide distributions (histograms or density plots) for uPTM-FetA and uL-FABP, stratified by KDIGO categories, plus medians [IQR] alongside means ± SD where skew is evident.

**Response:** As suggested, we have added histograms and density plots (**Fig 1**) to visualize the distribution of uPTM-FetA and uL-FABP across KDIGO risk categories. Given that these biomarkers exhibited significant skewness even after log-transformation, we have updated **Tables 1**, **2**, and **3** to report medians and interquartile ranges [IQR] alongside means and standard deviations (± SD). This ensures a transparent and accurate representation of the data distribution.

• Some table entries appear internally inconsistent or potentially mislabeled (e.g., units/layout for uPCR and the header “N† Number estimated”). Please standardize units and footnotes, and ensure alignment between text and tables.

**Response:** We fixed the points the reviewer pointed out.
• Where you state “no patients” had high uL-FABP in lower categories, explicitly report denominators; consider confidence intervals for proportions to show uncertainty.
**Response:** We have updated the Results section to explicitly report denominators (n/N) and 95% confidence intervals (CIs) for all proportions, including those where zero observations were recorded. This provides a clearer indication of the statistical uncertainty and strengthens our comparative analysis between uPTM-FetA and uL-FABP in early-stage risk categories.

**Statistics**
• Add a primary model specification (variables, transformation, and the clinical/biological rationale) before looking at univariate p-values to reduce data-driven selection.

**Response:** We fully agree with the importance of a hypothesis-driven approach to model building to avoid the pitfalls of data-driven variable selection. Accordingly, we have clarified in the Methods section that our primary multivariable model (Model 2) was predefined based on established clinical and biological rationales (e.g., age, sex, and known metabolic risk factors) rather than being selected solely based on univariate p-values.

• Report effect sizes for uPTM-FetA as continuous (per SD increase, per doubling if log-scaled) in addition to the binary threshold.

**Response:** To complement our binary threshold analysis, we have now performed a multivariable logistic regression using standardized values (Z-scores) for all continuous independent variables in Model 2. As shown in **S1 Table**, uPTM-FetA demonstrated a significant and independent association with DKD risk, with an odds ratio (OR) of 1.47 per 1-SD increase (95% CI: 1.03–2.24; p = 0.049). This confirms that uPTM-FetA maintains its independent clinical significance even when evaluated as a continuous measure.

• Add tests for linear trend across ordered KDIGO categories and a sensitivity analysis excluding extreme albuminuria (A3) or low eGFR (G4–G5), to probe whether associations persist in earlier disease.

**Response:** As requested, we performed two additional analyses to evaluate the utility of uPTM-FetA across the disease spectrum:

1. Sensitivity analysis for early-stage DKD: We conducted an analysis excluding patients with advanced disease (A3 and G4–G5 categories). In this model, we used raw clinical units to facilitate practical interpretation. The results strongly support the performance of uPTM-FetA in early-stage patients (**S3 Table**), where high uPTM-FetA (≥11.76 ng/mgCr) was associated with a 3.16-fold increase in the odds of DKD risk (OR: 3.16; 95% CI: 1.61–6.39; p < 0.01) after adjusting for other clinical factors.

2. Trend analysis: We performed the Jonckheere-Terpstra test to assess the linear trend across ordered KDIGO categories (1 to 4). This confirmed a significant progressive increase in uPTM-FetA levels with advancing risk stages (p for trend < 0.01, **Table 2**).

These findings collectively demonstrate that uPTM-FetA is a robust marker of renal risk, with significant clinical relevance starting from the early stages of disease.

**Discussion:**

• Strengths: First evaluation in a Japanese cohort; practical clinic setting.

**Response:** We have revised the Discussion to more clearly highlight the primary strengths of our study. Specifically, we emphasize that this is the first evaluation of uPTM-FetA within a Japanese cohort and that the study was conducted in a real-world, practical clinical setting, which enhances the external validity of our findings for similar outpatient populations.
• Limitations are acknowledged, but please explicitly add: single-center design; potential selection bias of outpatients; residual confounding (therapy intensity is a proxy for severity); single spot-urine measurement for both uACR and uPTM-FetA (intra-individual variability) — which you note for uACR and should also mention for uPTM-FetA.

**Response:** In accordance with the reviewer’s suggestion, we have significantly expanded the Limitations section. We now explicitly acknowledge the following constraints:

1. Study design: The single-center nature of the study may limit the generalizability of the results.

2. Selection bias: Potential bias inherent in an outpatient clinic population.

3. Residual confounding: The possibility that therapy intensity acts as a proxy for disease severity, potentially confounding the observed associations.

4. Measurement variability: The use of a single spot-urine measurement for both uACR and uPTM-FetA. We have clarified that intra-individual variability may affect both markers, extending the acknowledgment previously made for uACR to uPTM-FetA as well.

• Avoid implying superiority of uPTM-FetA over albuminuria without direct head-to-head discrimination/ reclassification metrics in the same modeling framework.

**Response:** We have carefully revised the manuscript to avoid any implications of uPTM-FetA’s superiority over albuminuria. All statements suggesting a comparative advantage have been removed or tempered. Instead, we now frame uPTM-FetA as a biomarker that provides complementary information regarding tubular stress, which may coexist with or precede glomerular changes. We explicitly state that further head-to-head discrimination and reclassification analyses within the same modeling framework would be required to claim diagnostic superiority.

**Transparency & compliance**
• Ethics/Registration: Adequately reported with approval number/date and UMIN ID.

**Response:** We appreciate the confirmation. The ethics approval details (Institutional Review Board approval number and date) and the clinical trial registration (UMIN ID) remain explicitly documented in the manuscript to ensure full ethical transparency.
• COI: Resolve the conflicting statements and present a single, accurate Competing Interests section consistent with PLOS policy (you already list specific companies and fees).

**Response:** We have resolved the inconsistencies regarding our conflict of interest disclosures. In accordance with PLOS policy, we have consolidated all financial relationships into a single, comprehensive "Competing Interests" section. This revised statement accurately reflects all relevant industry ties, including specific companies and the nature of the fees received by the authors.
• Data availability: Ensure S1 contains the full de-identified dataset with a variable dictionary sufficient to reproduce every figure/table (as claimed).

**Response:** We have ensured that the **S1 Dataset** contains the complete, row-level de-identified data. Furthermore, to facilitate independent verification and reproduction of our results, we have cross-referenced this dataset with **S1 Text** (Statistical Procedures), which serves as a detailed guide for reproducing every figure and table in the manuscript. We confirm that the provided information is sufficient for full data transparency as required by the journal.

**Minor comments (editorial/format)**
• Fix typographical errors (e.g., “Mesurements”) and ensure uniform units/abbreviation definitions at first mention.

**Response:** We have conducted a thorough proofreading of the manuscript and corrected all typographical errors, including the misspelling of “Measurements.” Furthermore, we have ensured that all units and abbreviations are defined at their first mention and used consistently throughout the text.
• Clarify whether “first-morning” or “random spot” urine was used (you note “morning on the day of visit” — specify fasting status and timing) and whether samples were processed identically.

**Response:** We have revised the manuscript to clarify whether first-morning or random spot urine samples were used. We now specify that urine samples were collected in the morning on the day of the visit, describe the fasting status and timing in detail, and confirm that all samples were processed using identical procedures.
• Add the exact SPSS version (you have “version 29.0”), and provide the code or detailed steps sufficient for replication (or upload analysis scripts as supplement).

**Response:** We have specified that IBM SPSS Statistics version 29.0 was used for the primary analyses. To ensure full transparency and reproducibility, we have provided a detailed, step-by-step description of our statistical workflow in **S1 Text**.

• Figures: consider adding confidence intervals to proportions in Figs 2–4 and include denominators on bars; supply accessible color palettes and ensure vector formats.

**Response:** We have revised our figures (now integrated as **Fig 6** in the revised manuscript) according to these suggestions. We have added 95% confidence intervals and denominators (n/N) to each bar to represent the precision of our estimates. Additionally, we have updated the figures using an accessible, color-blind friendly color palette and provided them in high-resolution PDF format (vector graphics) to ensure clarity and professional presentation.
• Ensure consistent reporting of decimals and SI units across all tables.

**Response:** We have revised all tables to ensure consistent reporting of decimal places and the use of SI units throughout the manuscript.
