# Supplementary material for: Association of urinary post-translationally modified fetuin-A fragments with diabetic kidney disease risk stratification in Japanese patients with type 2 diabetes
Source: PLoS One. 2026 Jul 2;21(7):e0353032. doi: 10.1371/journal.pone.0353032 (PMC13327179; doi:10.1371/journal.pone.0353032)
Supplement: Peer review history

# Editor report: Decision Letter 0

**DOI:** 10.1371/journal.pone.0353032.r001

**Authors:** Hesham Keryakos

2 Nov 2025

PONE-D-25-50146

Association between Diabetic Kidney Disease and Urinary excretion of post-translationally modified fetuin-A fragments in patients with type 2 diabetes.

PLOS ONE

Dear Dr.  MORI,

Thank you for submitting your manuscript to PLOS ONE. After careful consideration, we feel that it has merit but does not fully meet PLOS ONE’s publication criteria as it currently stands. Therefore, we invite you to submit a revised version of the manuscript that addresses the points raised during the review process.

Please submit your revised manuscript by Dec 17 2025 11:59PM. If you will need more time than this to complete your revisions, please reply to this message or contact the journal office at plosone@plos.org. When you're ready to submit your revision, log on to https://www.editorialmanager.com/pone/ and select the 'Submissions Needing Revision' folder to locate your manuscript file.

Please include the following items when submitting your revised manuscript:

- A rebuttal letter that responds to each point raised by the academic editor and reviewer(s). You should upload this letter as a separate file labeled 'Response to Reviewers'.
- A marked-up copy of your manuscript that highlights changes made to the original version. You should upload this as a separate file labeled 'Revised Manuscript with Track Changes'.
- An unmarked version of your revised paper without tracked changes. You should upload this as a separate file labeled 'Manuscript'.

If you would like to make changes to your financial disclosure, please include your updated statement in your cover letter. Guidelines for resubmitting your figure files are available below the reviewer comments at the end of this letter.

If applicable, we recommend that you deposit your laboratory protocols in protocols.io to enhance the reproducibility of your results. Protocols.io assigns your protocol its own identifier (DOI) so that it can be cited independently in the future. For instructions see: https://journals.plos.org/plosone/s/submission-guidelines#loc-laboratory-protocols. Additionally, PLOS ONE offers an option for publishing peer-reviewed Lab Protocol articles, which describe protocols hosted on protocols.io. Read more information on sharing protocols at https://plos.org/protocols?utm_medium=editorial-email&utm_source=authorletters&utm_campaign=protocols.

We look forward to receiving your revised manuscript.

Kind regards,

Hesham Kamal Habeeb Keryakos, Ph.D., M.D.

Academic Editor

PLOS ONE

Journal Requirements:

When submitting your revision, we need you to address these additional requirements.

1.Please ensure that your manuscript meets PLOS ONE's style requirements, including those for file naming. The PLOS ONE style templates can be found at

https://journals.plos.org/plosone/s/file?id=wjVg/PLOSOne_formatting_sample_main_body.pdf and

https://journals.plos.org/plosone/s/file?id=ba62/PLOSOne_formatting_sample_title_authors_affiliations.pdf

2. Thank you for stating the following in the Competing Interests section:

“Conflict of Interest

Hiroyuki Ito received lecture fees from Novo Nordisk Pharma Ltd., Eli Lilly Japan KK, Sanofi KK, Astellas Pharma, Kowa Company, Ltd., Taisho Pharmaceutical Co., Ltd., Sumitomo Pharma Co., Ltd., Boehringer Ingelheim, Daiichi Sankyo Company, Novartis Pharma KK, Takeda Pharmaceutical Company Ltd., MSD KK, Terumo Corporation, Mochida Pharmaceuticals, Teijin Pharma, Kissei Pharmaceuticals, Mitsubishi Tanabe Pharma Corporation, Sanwa Kagaku Kenkyusho, AstraZeneca KK, Kyowa Kirin Co. Ltd., Otsuka Pharmaceutical Co., Ltd., Bayer Yakuhin, Ltd., EA Pharma Co., Ltd., Ono Pharmaceutical Co., Ltd., and Viatris Inc., and received consulting fees from Becton, Dickinson and Company. Suzuko Matsumoto received lecture fees from Eli Lilly Japan KK, Novo Nordisk Pharma Ltd., Astellas Pharma, Kyowa Kirin Co., Ltd., and AstraZeneca KK. Hideyuki Inoue received lecture fees from Novartis Pharma KK, AstraZeneca KK, and Mochida Pharmaceuticals. Shinichi Antoku received lecture fees from Kyowa Kirin Co., Ltd., Sanofi KK, Taisho Pharmaceutical Co., Ltd., Daiichi Sankyo Company, Novo Nordisk Pharma Ltd., Novartis Pharma KK, AstraZeneca KK, and Otsuka Pharmaceutical Co., Ltd. Toshiko Mori received lecture fees from Novartis Pharma KK. Chizuko Yukawa has no conflict of interest.”

We note that you received funding from a commercial source: “Novo Nordisk Pharma Ltd”

Please provide an amended Competing Interests Statement that explicitly states this commercial funder, along with any other relevant declarations relating to employment, consultancy, patents, products in development, marketed products, etc.

Within this Competing Interests Statement, please confirm that this does not alter your adherence to all PLOS ONE policies on sharing data and materials by including the following statement: "This does not alter our adherence to PLOS ONE policies on sharing data and materials.” (as detailed online in our guide for authors http://journals.plos.org/plosone/s/competing-interests).  If there are restrictions on sharing of data and/or materials, please state these. Please note that we cannot proceed with consideration of your article until this information has been declared.

Please include your amended Competing Interests Statement within your cover letter. We will change the online submission form on your behalf.

3. We note that there is identifying data in the Supporting Information file < Supplement 1 (Deta Set).xlsx>. Due to the inclusion of these potentially identifying data, we have removed this file from your file inventory. Prior to sharing human research participant data, authors should consult with an ethics committee to ensure data are shared in accordance with participant consent and all applicable local laws.

Data sharing should never compromise participant privacy. It is therefore not appropriate to publicly share personally identifiable data on human research participants. The following are examples of data that should not be shared:

-Name, initials, physical address

-Ages more specific than whole numbers

-Internet protocol (IP) address

-Specific dates (birth dates, death dates, examination dates, etc.)

-Contact information such as phone number or email address

-Location data

-ID numbers that seem specific (long numbers, include initials, titled “Hospital ID”) rather than random (small numbers in numerical order)

Data that are not directly identifying may also be inappropriate to share, as in combination they can become identifying. For example, data collected from a small group of participants, vulnerable populations, or private groups should not be shared if they involve indirect identifiers (such as sex, ethnicity, location, etc.) that may risk the identification of study participants.

Additional guidance on preparing raw data for publication can be found in our Data Policy (https://journals.plos.org/plosone/s/data-availability#loc-human-research-participant-data-and-other-sensitive-data) and in the following article: http://www.bmj.com/content/340/bmj.c181.long.

Please remove or anonymize all personal information (<specific identifying information in file to be removed>), ensure that the data shared are in accordance with participant consent, and re-upload a fully anonymized data set. Please note that spreadsheet columns with personal information must be removed and not hidden as all hidden columns will appear in the published file.

4. If the reviewer comments include a recommendation to cite specific previously published works, please review and evaluate these publications to determine whether they are relevant and should be cited. There is no requirement to cite these works unless the editor has indicated otherwise.

Additional Editor Comments:

Summary

This cross-sectional study (n=219) explores whether urinary post-translationally modified fetuin-A fragments (uPTM-FetA) measured by a commercial ELISA are associated with KDIGO diabetic kidney disease (DKD) risk categories among Japanese outpatients with type 2 diabetes. The authors find higher prevalence of “high” uPTM-FetA (≥7.53 ng/mgCr, threshold imported from prior work) across higher DKD risk strata and an independent association with DKD categories 2–4 in multivariable models that exclude uL-FABP. Ethics approval and a UMIN registration are reported; analyses include rank-based tests and logistic regression with VIF<2 (no notable multicollinearity); KDIGO risk definitions and the 7.53 ng/mgCr cut-point are described in Methods.

Major comments

1. Cross-sectional design and causal language

The manuscript occasionally implies early detection/predictive potential. Given the design, please avoid inference about prediction or temporal precedence and reframe conclusions strictly as associations. Consider moving predictive language to “Future directions” and explicitly add a STROBE-consistent “Limitations” paragraph.

2. Choice and transportability of the uPTM-FetA cutoff (7.53 ng/mgCr)

The dichotomization threshold is imported from external cohorts. Provide a justification for transportability to this Japanese clinic population: distribution plots, within-sample ROC analysis vs KDIGO categories, and sensitivity analyses using uPTM-FetA as (a) a continuous predictor (with restricted cubic splines) and (b) alternative data-driven thresholds. Right now, heavy reliance on a single external cut-point risks misclassification and loss of information.

3. Model specification and potential mediator/collinearity handling

You present two multivariable models: one with uL-FABP and one excluding it, after noting VIF<2 for all variables. Please clarify the a priori causal framework: Is uL-FABP a competing biomarker, a mediator, or simply a correlated marker of tubular injury? If it’s a mediator, Model 1 may be “over-adjusted”; if not, exclusion in Model 2 risks omitted-variable bias. Pre-register or at least justify this strategy and report both sets fully (including calibrated probabilities and overall fit). Consider providing a DAG to clarify adjustment choices.

4. Outcome definition and risk-category amalgamation

The primary outcome collapses KDIGO categories 2–4 vs 1. Because these categories integrate both albuminuria and eGFR (with multiple pathways to “high risk”), the biology differs across 2, 3, and 4. Please (i) report separate models for each category vs 1, (ii) add trend tests across ordered risk categories, and (iii) provide partial effects plots for interpretability.

5. Confounding by medication and disease severity

Several drug classes (e.g., SGLT2i, RAAS blockade, urate-lowering agents) and comorbidities differ across groups and associate with DKD severity (see Tables; many differences are reported). At minimum, adjust your primary model for classes plausibly linked to tubular biomarkers and DKD severity, provide a medication-adjusted sensitivity analysis, and consider propensity-score or inverse probability weighting as robustness checks.

6. Measurement and pre-analytical detail for the ELISA

Add essential assay characteristics: lot numbers, intra/inter-assay CVs, LOD/LOQ, sample handling (first-morning vs random spot is stated; please add storage temp, freeze-thaw cycles), creatinine assays used for normalization, and whether batch effects were controlled (e.g., randomization of plates, inclusion of pooled QC). These are critical for biomarker papers and currently under-described.

7. Internal validity: multiple testing and model stability

Numerous univariate comparisons are presented. Please specify how you addressed multiplicity (e.g., false discovery rate) or emphasize that p-values are exploratory. For the logistic models, provide optimism-corrected performance (bootstrap) and calibration (e.g., calibration slope/intercept), even though the outcome is a categorical risk state.

8. Interpretation versus uL-FABP

The statement that uPTM-FetA “rises earlier than uL-FABP” is inferred from cross-sectional proportions (e.g., no high uL-FABP in lower DKD risk groups) rather than longitudinal change. Please temper this claim and reframe as “uPTM-FetA was more frequently elevated than uL-FABP in lower KDIGO risk states in this cross-sectional snapshot,” or demonstrate incremental discrimination (e.g., likelihood-ratio test for nested models, partial AUC) within this design.

9. COI/Data availability inconsistencies

The submission packet contains both a templated “NO authors have competing interests” statement and, elsewhere, detailed financial relationships; this must be reconciled to PLOS standards. Similarly, the Data Availability statement asserts that “All relevant data are within the manuscript and its Supporting Information files” — ensure the S1 dataset actually contains row-level, de-identified data and a codebook that reproduce all tables/figures.

Specific/section-by-section notes

Title/Abstract

• Clear scope. In the Conclusions, remove predictive framing (“early detection”) or qualify it as hypothesis-generating given the design.

• Include the study dates and setting in the abstract Methods for clarity (Edogawa Hospital; 2023-11-16 to 2024-02-26) to align with STROBE.

Introduction

• Nicely motivated. Consider briefly differentiating mechanistic pathways (glomerular vs tubular injury) to frame why uPTM-FetA might capture injury distinct from albuminuria and uL-FABP, and cite the KDIGO risk grid’s composite nature to justify stratified analyses.

Methods

• KDIGO risk categories and biomarker cut-points are well defined. Please correct small typos (“Mesurements”), and add analytic decisions: handling of outliers, missingness, transformations (log-uPTM-FetA, log-uACR), and model diagnostics.

• Ethics and registration are appropriately documented (committee, number/date, UMIN ID) — good

• Explicitly state the eGFR formula (Japanese Society of Nephrology) and lab methods; you currently reference the formula but not its equation or creatinine assay traceability

Results

• Provide distributions (histograms or density plots) for uPTM-FetA and uL-FABP, stratified by KDIGO categories, plus medians [IQR] alongside means ± SD where skew is evident.

• Some table entries appear internally inconsistent or potentially mislabeled (e.g., units/layout for uPCR and the header “N† Number estimated”). Please standardize units and footnotes, and ensure alignment between text and tables.

• Where you state “no patients” had high uL-FABP in lower categories, explicitly report denominators; consider confidence intervals for proportions to show uncertainty.

Statistics

• Add a primary model specification (variables, transformation, and the clinical/biological rationale) before looking at univariate p-values to reduce data-driven selection.

• Report effect sizes for uPTM-FetA as continuous (per SD increase, per doubling if log-scaled) in addition to the binary threshold.

• Add tests for linear trend across ordered KDIGO categories and a sensitivity analysis excluding extreme albuminuria (A3) or low eGFR (G4–G5), to probe whether associations persist in earlier disease.

Discussion

• Strengths: First evaluation in a Japanese cohort; practical clinic setting.

• Limitations are acknowledged, but please explicitly add: single-center design; potential selection bias of outpatients; residual confounding (therapy intensity is a proxy for severity); single spot-urine measurement for both uACR and uPTM-FetA (intra-individual variability) — which you note for uACR and should also mention for uPTM-FetA.

• Avoid implying superiority of uPTM-FetA over albuminuria without direct head-to-head discrimination/ reclassification metrics in the same modeling framework.

Transparency & compliance

• Ethics/Registration: Adequately reported with approval number/date and UMIN ID.

• COI: Resolve the conflicting statements and present a single, accurate Competing Interests section consistent with PLOS policy (you already list specific companies and fees).

• Data availability: Ensure S1 contains the full de-identified dataset with a variable dictionary sufficient to reproduce every figure/table (as claimed).

Minor comments (editorial/format)

• Fix typographical errors (e.g., “Mesurements”) and ensure uniform units/abbreviation definitions at first mention.

• Clarify whether “first-morning” or “random spot” urine was used (you note “morning on the day of visit” — specify fasting status and timing) and whether samples were processed identically.

• Add the exact SPSS version (you have “version 29.0”), and provide the code or detailed steps sufficient for replication (or upload analysis scripts as supplement).

• Figures: consider adding confidence intervals to proportions in Figs 2–4 and include denominators on bars; supply accessible color palettes and ensure vector formats.

• Ensure consistent reporting of decimals and SI units across all tables.

Recommendation

Major revision. The study addresses an important clinical gap and is suitable for PLOS ONE’s scope if framed as an associative analysis. With clarified modeling, stronger assay/reporting details, resolved COI/data-availability issues, and tempered causal claims, the manuscript would be substantially strengthened.

[Note: HTML markup is below. Please do not edit.]

[NOTE: If reviewer comments were submitted as an attachment file, they will be attached to this email and accessible via the submission site. Please log into your account, locate the manuscript record, and check for the action link "View Attachments". If this link does not appear, there are no attachment files.]

To ensure your figures meet our technical requirements, please review our figure guidelines: https://journals.plos.org/plosone/s/figures

You may also use PLOS’s free figure tool, NAAS, to help you prepare publication quality figures: https://journals.plos.org/plosone/s/figures#loc-tools-for-figure-preparation.

NAAS will assess whether your figures meet our technical requirements by comparing each figure against our figure specifications.

# Author comment: Author response to Decision Letter 1

**DOI:** 10.1371/journal.pone.0353032.r002

15 Feb 2026

Responses to Academic Editor and Reviewers

Journal Requirements

Requirement 1 (Style requirements): Please ensure that your manuscript meets PLOS ONE's style requirements, including those for file naming.

Response: We have thoroughly reviewed the PLOS ONE style templates and formatted both the manuscript and file names accordingly.

Requirement 2 (Competing Interests): We note that you received funding from a commercial source: “Novo Nordisk Pharma Ltd”. Please provide an amended Competing Interests Statement...

Response: We would like to clarify that we did not receive any research funding or study support from Novo Nordisk Pharma Ltd. Our relationship with Novo Nordisk Pharma Ltd. is limited to the receipt of lecture fees. We believe that the reference to “funding from a commercial source” may have resulted from a misunderstanding of our initial disclosure. We would appreciate your consideration of the following amended Competing Interests Statement:

Hiroyuki Ito received lecture fees from Novo Nordisk Pharma Ltd., Eli Lilly Japan KK, Sanofi KK, Astellas Pharma, Kowa Company, Ltd., Taisho Pharmaceutical Co., Ltd., Sumitomo Pharma Co., Ltd., Boehringer Ingelheim, Daiichi Sankyo Company, Novartis Pharma KK, Takeda Pharmaceutical Company Ltd., MSD KK, Terumo Corporation, Mochida Pharmaceuticals, Teijin Pharma, Kissei Pharmaceuticals, Mitsubishi Tanabe Pharma Corporation, Sanwa Kagaku Kenkyusho, AstraZeneca KK, Kyowa Kirin Co. Ltd., Otsuka Pharmaceutical Co., Ltd., Bayer Yakuhin, Ltd., EA Pharma Co., Ltd., Ono Pharmaceutical Co., Ltd., and Viatris Inc., and received consulting fees from Becton, Dickinson and Company.

Suzuko Matsumoto received lecture fees from Eli Lilly Japan KK, Novo Nordisk Pharma Ltd., Astellas Pharma, Kyowa Kirin Co., Ltd., and AstraZeneca KK.

Hideyuki Inoue received lecture fees from Novartis Pharma KK, AstraZeneca KK, and Mochida Pharmaceuticals.

Shinichi Antoku received lecture fees from Kyowa Kirin Co., Ltd., Sanofi KK, Taisho Pharmaceutical Co., Ltd., Daiichi Sankyo Company, Novo Nordisk Pharma Ltd., Novartis Pharma KK, AstraZeneca KK, and Otsuka Pharmaceutical Co., Ltd.

Toshiko Mori received lecture fees from Novartis Pharma KK.

Chizuko Yukawa has no conflict of interest.

This does not alter our adherence to PLOS ONE policies on sharing data and materials.

Requirement 3 (Data Anonymization): We note that there is identifying data in the Supporting Information file < Supplement 1 (Deta Set).xlsx>... Please remove or anonymize all personal information.

Response: In accordance with this requirement, we have removed all potentially identifying information from the S1 Dataset to ensure participant privacy. The fully anonymized dataset has been re-uploaded as part of this revision.

Requirement 4 (Citing references): If the reviewer comments include a recommendation to cite specific previously published works, please review and evaluate these publications to determine whether they are relevant and should be cited. There is no requirement to cite these works unless the editor has indicated otherwise.

Response: Thank you for this guidance. While no specific publications were recommended by the reviewers, we have thoroughly reviewed recent literature pertaining to uPTM-FetA, DKD biomarkers, and tubular injury markers. Consequently, we have incorporated several relevant studies into the revised manuscript to better contextualize our findings within the current landscape of diabetic kidney disease research. We believe these additional citations have strengthened the theoretical framework and clinical relevance of our study.

Response to Academic Editor and Reviewers

Academic Editor Comments

Major comments

Comment 1 (Cross-sectional design and causal language): The manuscript occasionally implies early detection/predictive potential. Given the design, please avoid inference about prediction or temporal precedence and reframe conclusions strictly as associations. Consider moving predictive language to “Future directions” and explicitly add a STROBE-consistent “Limitations” paragraph.

Response: We agree with the Editor’s assessment. Given the cross-sectional nature of this study, we have carefully revised the manuscript to avoid language implying causality or predictive capacity (e.g., replacing "predict" with "is associated with"). As suggested, speculative discussion regarding the predictive potential of uPTM-FetA has been moved to the "Future directions" section. Furthermore, we have added a dedicated "Limitations" subsection within the Discussion to explicitly address the inherent constraints of our study design, ensuring full compliance with STROBE guidelines.

Comment 2 (Choice and transportability of the uPTM-FetA cutoff (7.53 ng/mgCr)): The dichotomization threshold is imported from external cohorts. Provide a justification for transportability to this Japanese clinic population: distribution plots, within-sample ROC analysis vs KDIGO categories, and sensitivity analyses using uPTM-FetA as (a) a continuous predictor (with restricted cubic splines) and (b) alternative data-driven thresholds. Right now, heavy reliance on a single external cut-point risks misclassification and loss of information.

Response: We agree that a robust justification for the threshold is essential for the transportability of the biomarker to our specific study population. Accordingly, we have performed the following additional analyses:

1. Distribution plots: To visualize the data structure, we have included histograms of uPTM-FetA levels (both raw and log-transformed) in the revised manuscript (Fig 1A and 1B). Additionally, a density plot stratified by DKD-risk category has been added to show the shift in uPTM-FetA distribution (Fig 1C).

2. Within-sample ROC Analysis: We conducted a ROC analysis to evaluate the diagnostic performance of uPTM-FetA in identifying DKD-risk categories 2+3+4. The area under the curve (AUC) was 0.64. Based on the Youden index, the optimal data-driven cutoff for this cohort was identified as 11.76 ng/mgCr (Sensitivity: 0.66, Specificity: 0.66; Fig 4). Consequently, we have adopted this specific cutoff value (11.76 ng/mgCr) for our binary analyses in this study. The discrepancy between this data-driven threshold and previously reported values (e.g., 7.53 ng/mgCr) is now addressed in the Discussion section.

3. Continuous variable analysis (Restricted cubic splines): To avoid potential information loss from dichotomization, we performed sensitivity analyses using uPTM-FetA as a continuous variable. The restricted cubic spline (RCS) model confirmed a significant non-linear association between uPTM-FetA levels and the increased odds of higher DKD-risk categories, reinforcing the robustness of our findings (Fig 3A).

Comment 3 (Model specification and potential mediator/collinearity handling): You present two multivariable models: one with uL-FABP and one excluding it, after noting VIF<2 for all variables. Please clarify the a priori causal framework: Is uL-FABP a competing biomarker, a mediator, or simply a correlated marker of tubular injury? If it’s a mediator, Model 1 may be “over-adjusted”; if not, exclusion in Model 2 risks omitted-variable bias. Pre-register or at least justify this strategy and report both sets fully (including calibrated probabilities and overall fit). Consider providing a DAG to clarify adjustment choices.

Response: We appreciate this insightful request to clarify our conceptual framework. We consider uL-FABP and uPTM-FetA to be correlated markers reflecting distinct, though potentially overlapping, pathological pathways of tubular injury. We do not hypothesize that uL-FABP acts as a direct mediator in the causal pathway between uPTM-FetA and DKD severity.

To address the risk of over-adjustment while ensuring robust results, we have adopted the following strategy:

1. Primary model (Model 2): We designate Model 2 (unadjusted for uL-FABP) as our primary model to evaluate the independent association of uPTM-FetA with DKD risk categories.

2. Sensitivity analysis (Model 1): Model 1 (adjusted for uL-FABP) is presented as a sensitivity analysis. This model confirms that the association between uPTM-FetA and DKD-risk remains statistically significant even after accounting for uL-FABP, a well-established marker of tubular stress. This demonstrates that uPTM-FetA provides distinct clinical information independent of uL-FABP.

We have added a statement to the Methods section to clarify this a priori modeling framework. Furthermore, the physiological and clinical differences between uPTM-FetA and uL-FABP are now elaborated upon in the Discussion section. We believe this strategy effectively addresses the concern of omitted-variable bias while avoiding the pitfalls of over-adjustment.

Comment 4 (Outcome definition and risk-category amalgamation): The primary outcome collapses KDIGO categories 2–4 vs 1. Because these categories integrate both albuminuria and eGFR (with multiple pathways to “high risk”), the biology differs across 2, 3, and 4. Please (i) report separate models for each category vs 1, (ii) add trend tests across ordered risk categories, and (iii) provide partial effects plots for interpretability.

Response: We acknowledge that biological pathophysiology may differ across KDIGO risk categories 2, 3, and 4. As suggested, we have addressed this by performing more granular analyses to evaluate the association of uPTM-FetA across the full spectrum of DKD risk:

1. Multinomial logistic regression: To evaluate each category separately, we performed multinomial logistic regression using KDIGO category 1 as the reference group. The odds ratios (ORs) for uPTM-FetA exposure for categories 2, 3, and 4 are now reported individually in S2 Table.

2. Trend test: To assess the dose-response relationship, we conducted the Jonckheere–Terpstra test for linear trends across the ordered KDIGO risk categories (1 to 4). This analysis confirmed a highly significant progressive increase in uPTM-FetA levels associated with advancing risk stages (p for trend < 0.01).

3. Partial effects plots: To enhance interpretability, we have included a partial effects plot (Fig 3B). This figure illustrates the predicted probabilities of DKD-risk (categories 2+3+4) across the range of uPTM-FetA values, after adjusting for potential confounders.

Comment 5 (Confounding by medication and disease severity): Several drug classes (e.g., SGLT2i, RAAS blockade, urate-lowering agents) and comorbidities differ across groups and associate with DKD severity (see Tables; many differences are reported). At minimum, adjust your primary model for classes plausibly linked to tubular biomarkers and DKD severity, provide a medication-adjusted sensitivity analysis, and consider propensity-score or inverse probability weighting as robustness checks.

Response: We agree that medications affecting renal hemodynamics or DKD progression could act as potential confounders. To address this, we performed a medication-adjusted sensitivity analysis as requested.

We constructed an extended multivariable model that includes the use of SGLT2 inhibitors, GLP-1 receptor agonists, RAAS inhibitors, calcium channel blockers, and urate-lowering agents as additional independent variables (S4 Table). The results demonstrated that even after rigorous adjustment for these therapeutic classes, there were no significant changes in the direction, magnitude, or statistical significance of the odds ratio for high uPTM-FetA. These findings confirm the independent association of uPTM-FetA with DKD risk and underline the robustness of our primary model against potential confounding by medication use.

Comment 6 (Measurement and pre-analytical detail for the ELISA): Add essential assay characteristics: lot numbers, intra/inter-assay CVs, LOD/LOQ, sample handling (first-morning vs random spot is stated; please add storage temp, freeze-thaw cycles), creatinine assays used for normalization, and whether batch effects were controlled (e.g., randomization of plates, inclusion of pooled QC). These are critical for biomarker papers and currently under-described.

Response: We fully recognize the importance of providing detailed assay characteristics for the transparency and reliability of biomarker studies. In response to this comment, we have extensively updated the Methods section to include the following technical specifications:

1. Assay performance: We have specified the lot numbers of the ELISA kits used, the intra- and inter-assay coefficients of variation (CVs), and the limit of detection (LOD) and limit of quantification (LOQ).

2. Sample handling and storage: We have clarified that spot urine samples were stored at -80°C immediately after collection and processing. We also explicitly stated that samples underwent no more than one freeze-thaw cycle prior to analysis.

3. Creatinine normalization: Details regarding the enzymatic assay used for urinary creatinine measurement and its traceability have been added.

4. Quality and batch control: To minimize batch effects, samples were randomized across plates, and internal pooled quality control (QC) samples were included in each run to monitor assay stability.

These additions ensure that our methodology adheres to the rigorous standards required for biomarker validation.

Comment 7 (Internal validity: multiple testing and model stability): Numerous univariate comparisons are presented. Please specify how you addressed multiplicity (e.g., false discovery rate) or emphasize that p-values are exploratory. For the logistic models, provide optimism-corrected performance (bootstrap) and calibration (e.g., calibration slope/intercept), even though the outcome is a categorical risk state.

Response: We have addressed the concerns regarding internal validity and model stability through the following two key revisions:

1. Multiplicity: We have explicitly stated in the Statistical Analysis section that this is an exploratory study. Consequently, p-values were not adjusted for multiple comparisons, and our findings should be interpreted as suggestive of associations intended to generate hypotheses for future research.

2. Model stability and internal validation: To address the potential for overfitting and to evaluate the internal validity of Model 2, we performed a rigorous internal validation using a nonparametric bootstrap resampling procedure (1,000 iterations).

- Discriminative ability: While the apparent AUC was 0.765, the optimism-corrected AUC was 0.731, indicating satisfactory discriminative performance even after accounting for potential overfitting.

- Calibration: The calibration slope was 0.815 (approaching the ideal value of 1.0), and the corrected Nagelkerke R² was 0.193.

- Visualization: To visually supplement these findings, we have generated a calibration plot (Fig 5), which demonstrates excellent agreement between the predicted and observed probabilities of DKD-risk categories across the entire spectrum.

2. Model stability: To address the potential for overfitting and to evaluate the internal validity of Model 2, we performed an internal validation using a bootstrap resampling procedure (1,000 iterations). The validation results demonstrated the stability and reliability of the model. While the apparent AUC was 0.765, the optimism-corrected AUC was 0.731, indicating a satisfactory discriminative ability even after adjusting for potential overfitting. Furthermore, the calibration slope was 0.815, which is close to the ideal value of 1.0, and the corrected Nagelkerke R2 was 0.193. To visually supplement these findings, we have also generated a calibration plot (Fig 5), which shows an excellent agreement between the predicted and observed probabilities across the entire risk spectrum.

These validation results confirm that the association between high uPTM-FetA and DKD risk is robust and that our model maintains stable performance for clinical risk assessment. These details have been incorporated into the Methods and Results sections.

Comment 8 (Interpretation versus uL-FABP): The statement that uPTM-FetA “rises earlier than uL-FABP” is inferred from cross-sectional proportions (e.g., no high uL-FABP in lower DKD risk groups) rather than longitudinal change. Please temper this claim and reframe a

Attachment Submitted filename: Response to Reviewers.docx

# Peer review: Decision Letter 1

**DOI:** 10.1371/journal.pone.0353032.r003

**Authors:** Hesham Keryakos, Tatsuo Shimosawa

18 Jun 2026

<p>Association of urinary post-translationally modified fetuin-A fragments with diabetic kidney disease risk stratification in Japanese patients with type 2 diabetes

PONE-D-25-50146R1

Dear Dr. Ito,

We’re pleased to inform you that your manuscript has been judged scientifically suitable for publication and will be formally accepted for publication once it meets all outstanding technical requirements.

Within one week, you’ll receive an e-mail detailing the required amendments. When these have been addressed, you’ll receive a formal acceptance letter and your manuscript will be scheduled for publication.

An invoice will be generated when your article is formally accepted. Please note, if your institution has a publishing partnership with PLOS and your article meets the relevant criteria, all or part of your publication costs will be covered. Please make sure your user information is up-to-date by logging into Editorial Manager at Editorial Manager® and clicking the ‘Update My Information' link at the top of the page. For questions related to billing, please contact billing support.

If your institution or institutions have a press office, please notify them about your upcoming paper to help maximize its impact. If they’ll be preparing press materials, please inform our press team as soon as possible -- no later than 48 hours after receiving the formal acceptance. Your manuscript will remain under strict press embargo until 2 pm Eastern Time on the date of publication. For more information, please contact onepress@plos.org.

Kind regards,

Tatsuo Shimosawa, M.D., Ph.D.

Academic Editor

PLOS One

Additional Editor Comments (optional):

Reviewers' comments:

Reviewer's Responses to Questions

**Comments to the Author**

1. If the authors have adequately addressed your comments raised in a previous round of review and you feel that this manuscript is now acceptable for publication, you may indicate that here to bypass the “Comments to the Author” section, enter your conflict of interest statement in the “Confidential to Editor” section, and submit your "Accept" recommendation.

Reviewer #1: All comments have been addressed

**********

2. Is the manuscript technically sound, and do the data support the conclusions?

The manuscript must describe a technically sound piece of scientific research with data that supports the conclusions. Experiments must have been conducted rigorously, with appropriate controls, replication, and sample sizes. The conclusions must be drawn appropriately based on the data presented.

Reviewer #1: Yes

**********

3. Has the statistical analysis been performed appropriately and rigorously?

Reviewer #1: I Don't Know

**********

4. Have the authors made all data underlying the findings in their manuscript fully available?

The PLOS Data policy requires authors to make all data underlying the findings described in their manuscript fully available without restriction, with rare exception (please refer to the Data Availability Statement in the manuscript PDF file). The data should be provided as part of the manuscript or its supporting information, or deposited to a public repository. For example, in addition to summary statistics, the data points behind means, medians and variance measures should be available. If there are restrictions on publicly sharing data—e.g. participant privacy or use of data from a third party—those must be specified.

Reviewer #1: Yes

**********

5. Is the manuscript presented in an intelligible fashion and written in standard English?

PLOS ONE does not copyedit accepted manuscripts, so the language in submitted articles must be clear, correct, and unambiguous. Any typographical or grammatical errors should be corrected at revision, so please note any specific errors here.

Reviewer #1: No

**********

6. Review Comments to the Author

Please use the space provided to explain your answers to the questions above. You may also include additional comments for the author, including concerns about dual publication, research ethics, or publication ethics. (Please upload your review as an attachment if it exceeds 20,000 characters)

Reviewer #1: Authors have made changes while revising the manuscript. However, if a professional biostatistician certificate is also attached that robust review of all the statistics related data done would be more appropriate.

**********

7. PLOS authors have the option to publish the peer review history of their article (what does this mean?). If published, this will include your full peer review and any attached files.

If you choose “no”, your identity will remain anonymous but your review may still be made public.

**Do you want your identity to be public for this peer review?**  For information about this choice, including consent withdrawal, please see our Privacy Policy.

Reviewer #1: **Yes:** Jamshed Akhtar

**********

# Editor report: Acceptance letter

**DOI:** 10.1371/journal.pone.0353032.r004

**Authors:** Hesham Keryakos, Tatsuo Shimosawa

PONE-D-25-50146R1

PLOS One

Dear Dr. Ito,

I'm pleased to inform you that your manuscript has been deemed suitable for publication in PLOS One. Congratulations! Your manuscript is now being handed over to our production team.

At this stage, our production department will prepare your paper for publication. This includes ensuring the following:

* All references, tables, and figures are properly cited

* All relevant supporting information is included in the manuscript submission,

* There are no issues that prevent the paper from being properly typeset

You will receive further instructions from the production team, including instructions on how to review your proof when it is ready. Please keep in mind that we are working through a large volume of accepted articles, so please give us a few days to review your paper and let you know the next and final steps.

Lastly, if your institution or institutions have a press office, please let them know about your upcoming paper now to help maximize its impact. If they'll be preparing press materials, please inform our press team within the next 48 hours. Your manuscript will remain under strict press embargo until 2 pm Eastern Time on the date of publication. For more information, please contact onepress@plos.org.

You will receive an invoice from PLOS for your publication fee after your manuscript has reached the completed accept phase. If you receive an email requesting payment before acceptance or for any other service, this may be a phishing scheme. Learn how to identify phishing emails and protect your accounts at https://explore.plos.org/phishing.

If we can help with anything else, please email us at customercare@plos.org.

Thank you for submitting your work to PLOS ONE and supporting open access.

Kind regards,

PLOS ONE Editorial Office Staff

on behalf of

Prof. Tatsuo Shimosawa

Academic Editor

PLOS One
